# Risk of Venous Thromboembolic Events After Surgery for Cancer

**DOI:** 10.1001/jamanetworkopen.2023.54352

**Published:** 2024-02-02

**Authors:** Johan Björklund, Juhana Rautiola, Renata Zelic, Gustaf Edgren, Matteo Bottai, Magnus Nilsson, Per Henrik Vincent, Hanna Fredholm, Henrik Falconer, Annika Sjövall, Per J. Nilsson, Peter Wiklund, Markus Aly, Olof Akre

**Affiliations:** 1 Department of Molecular Medicine and Surgery, Karolinska Institutet, Stockholm, Sweden; 2 Department of Pelvic Cancer, Karolinska University Hospital, Stockholm, Sweden; 3 Clinical Epidemiology Division, Department of Medicine, Karolinska Institutet, Stockholm, Sweden; 4 Department of Cardiology, Södersjukhuset, Stockholm, Sweden; 5 Division of Biostatistics, Institute of Environmental Medicine, Karolinska Institutet, Stockholm, Sweden; 6 Division of Surgery and Oncology, Department of Clinical Science, Intervention and Technology, Karolinska Institutet, Stockholm, Sweden; 7 Department of Upper Abdominal Diseases, Karolinska University Hospital, Stockholm, Sweden; 8 Department of Molecular Medicine and Surgery, Karolinska Institutet, Stockholm, Sweden; 9 Department of Women’s and Children’s Health, Karolinska Institutet, Stockholm, Sweden; 10 Department of Urology, Icahn School of Medicine at Mount Sinai, New York, New York

## Abstract

**Importance:**

The risks and benefits of thromboprophylaxis therapy after cancer surgery are debated. Studies that determine thrombosis risk after cancer surgery with high accuracy are needed.

**Objectives:**

To evaluate 1-year risk of venous thromboembolic events after major cancer surgery and how these events vary over time.

**Design, Setting, and Participants:**

This register-based retrospective observational matched cohort study included data on the full population of Sweden between 1998 and 2016. All patients who underwent major surgery for cancer of the bladder, breast, colon or rectum, gynecologic organs, kidney and upper urothelial tract, lung, prostate, or gastroesophageal tract were matched in a 1:10 ratio with cancer-free members of the general population on year of birth, sex, and county of residence. Data were analyzed from February 13 to December 5, 2023.

**Exposure:**

Major surgery for cancer.

**Main Outcomes and Measures:**

The main outcome was incidence of venous thromboembolic events within 1 year after the surgery. Crude absolute risks and risk differences of events within 1 year and adjusted time-dependent cause-specific hazard ratios (HRs) of postdischarge events were calculated.

**Results:**

A total of 432 218 patients with cancer (median age, 67 years [IQR, 58-75 years]; 68.7% women) and 4 009 343 cancer-free comparators (median age, 66 years [IQR, 57-74 years]; 69.3% women) were included in the study. The crude 1-year cumulative risk of pulmonary embolism was higher among the cancer surgery population for all cancers, with the following absolute risk differences: for bladder cancer, 2.69 percentage points (95% CI, 2.33-3.05 percentage points); for breast cancer, 0.59 percentage points (95% CI 0.55-0.63 percentage points); for colorectal cancer, 1.57 percentage points (95% CI, 1.50-1.65 percentage points); for gynecologic organ cancer, 1.32 percentage points (95% CI, 1.22-1.41 percentage points); for kidney and upper urinary tract cancer, 1.38 percentage points (95% CI, 1.21-1.55 percentage points); for lung cancer, 2.61 percentage points (95% CI, 2.34-2.89 percentage points); for gastroesophageal cancer, 2.13 percentage points (95% CI, 1.89-2.38 percentage points); and for prostate cancer, 0.57 percentage points (95% CI, 0.49-0.66 percentage points). The cause-specific HR of pulmonary embolism comparing patients who underwent cancer surgery with matched comparators peaked just after discharge and generally plateaued 60 to 90 days later. At 30 days after surgery, the HR was 10 to 30 times higher than in the comparison cohort for all cancers except breast cancer (colorectal cancer: HR, 9.18 [95% CI, 8.03-10.50]; lung cancer: HR, 25.66 [95% CI, 17.41-37.84]; breast cancer: HR, 5.18 [95% CI, 4.45-6.05]). The hazards subsided but never reached the level of the comparison cohort except for prostate cancer. Similar results were observed for deep vein thrombosis.

**Conclusions and Relevance:**

This cohort study found an increased rate of venous thromboembolism associated with cancer surgery. The risk persisted for about 2 to 4 months postoperatively but varied between cancer types. The increased rate is likely explained by the underlying cancer disease and adjuvant treatments. The results highlight the need for individualized venous thromboembolism risk evaluation and prophylaxis regimens for patients undergoing different surgery for different cancers.

## Key Points

**Question:**

Is major cancer surgery associated with an increased risk of venous thromboembolism after surgery?

**Findings:**

This population-based nationwide cohort study comparing 432 218 patients who underwent major surgery for 8 cancer types with 4 009 343 matched general population comparators found a statistically significant increase in 1-year cumulative risk of venous thromboembolism. The initial increase associated with the surgery was followed by a plateau that varied by cancer type.

**Meaning:**

These findings suggest that there is an increased risk of venous thromboembolism associated with cancer surgery, which varies both in magnitude and temporality by cancer type.

## Teaser

This cohort study evaluates the 1-year risk of venous thromboembolic events after major cancer surgery and how these events vary over time.

## Introduction

Major surgery and cancer are both risk factors for venous thromboembolism,^1,2^ causing considerable mortality and morbidity.^3^ Studies suggest that around 2% of patients who undergo cancer surgery develop clinically significant venous thromboembolism, accounting for around 50% of the early postoperative mortality.^4^

To lower the risk of venous thromboembolic events, prophylactic administration of low molecular-weight heparin and other anticoagulants is clinical routine for many surgical procedures.^5^ However, the timing, duration, dosage, and choice of prophylaxis is based on limited data.

In many clinical guidelines, extended prophylaxis of 28 days^6,7,8^ is recommended for patients undergoing cancer surgery.^5,9,10^ However, such recommendations are not based on precise estimates of disease-specific excess risk of thromboembolic events.^11,12^ Estimation of disease-specific risk is difficult and requires large surgical cohorts, and comparison populations are rarely available. We used nationwide and population-based health care and population databases to provide estimates of the risk of venous thromboembolic events and their temporal patterns among patients undergoing major surgery for different cancers.

## Methods

### Data Sources

In this cohort study, we used multiple Swedish nationwide registers linked via the personal identification number that is assigned to each Swedish resident. The Swedish National Patient Register covers all inpatient care in Sweden from 1987 onward, and specialist outpatient care from 1997 onward.^13,14^ The Patient Register records all diagnoses using the Swedish version of the *International Classification of Diseases, Ninth Revision* (*ICD-9*) (1987-1996) and the *International Statistical Classification of Diseases and Related Health Problems, Tenth Revision* (*ICD-10*) (1997 onward) and procedures using the National Classification For Operations^15^ (1987-1996) and an adapted version of the NOMESCO (Nordic Medico-Statistical Committee) Classification of Surgical Procedures^16^ (1997 onward). Since 1952, all dates and causes of death are recorded in the Cause of Death Register.^17^ The Swedish Total Population Register has been available since 1968 and contains demographic information on all persons born or residing in Sweden.^18^ The Swedish National Cancer Register contains information on all cancers diagnosed in Sweden since 1958 and reporting is mandated by law.^19^ This study was approved by the Regional Ethics Committee in Stockholm. The need for informed consent was waived by the ethics committee because the data used for analyses were pseudonymized. This study followed the Strengthening the Reporting of Observational Studies in Epidemiology (STROBE) reporting guideline.

### Cancer Surgery Cohort

The cancer surgery cohort included all patients who underwent major surgery for cancer of the urinary bladder, breast, colon or rectum, gynecologic organs, kidney or upper urinary tract urothelial cancer, lung, prostate, or stomach or esophagus from 1987 to 2016, as recorded in the Swedish National Patient Register. Detailed inclusion criteria are presented in eTable 1 in Supplement 1. We started follow-up on the date of the index cancer surgery, defined as the date of admission for the surgery. For each patient, we retrieved all other diagnoses and procedures within 1 year before and after the index date. Patients with a diagnosis of a venous thromboembolic event 1 year prior to the index date were excluded.

### Matched Comparison Cohort

For each patient in the cancer surgery cohort, we randomly selected 10 individuals from the Swedish Total Population Register, matched by year of birth, sex, and county of residence. We assigned the index date of the corresponding patient in the cancer surgery cohort to each individual in the comparison cohort and collected pre- and post-index date data as described for the cancer surgery cohort. The matched comparators were cancer free before the index date according to the Cancer Register but could develop cancer during the follow-up.

### Outcome Definition and Follow-Up

The study population was followed using the Swedish Patient Register and the Cause of Death Register for the occurrence of first nonfatal or fatal pulmonary embolism or deep vein thrombosis within 1 year after the index date. All types of venous thromboembolic events were included, including subsegmental pulmonary embolism and deep venous thromboembolism in the calf, as they were not differentiated in the diagnostic data. Pulmonary embolism was identified by the following codes: 415 (*ICD-9*) or I26 (*ICD-10*). Deep vein thrombosis was defined by the following codes: 451 to 453 (*ICD-9*) and I80 to I82 (*ICD-10*). The Swedish Patient Register has an overall positive predictive value of 85% to 95% for most diagnoses,^13^ but the specific validity of diagnoses of venous thromboembolism has not been assessed.

Venous thromboembolic events occurring in the hospital may be different than those occurring after discharge, and the exact date of an event during hospitalization was not known. Therefore, we separated outcomes during the index hospitalization and after discharge for all nondescriptive analyses. For the analysis during the index hospitalization, the individuals in the matched comparison cohort were assigned the same length of hospitalization as their matched patients in the cancer surgery cohort.

### Statistical Analysis

Statistical analysis was conducted from February 13 to December 5, 2023. We calculated crude absolute risks (ARs) of pulmonary embolism and deep vein thrombosis for the cancer surgery and matched comparison cohorts at 30, 90, and 365 days from the index date, as well as the absolute risk difference (ARD) with the corresponding 95% CIs. Patients who were still hospitalized at these time points, and their corresponding comparators, were excluded. Recurring events were not counted.

We used logistic regression to estimate odds ratios and 95% CIs for the outcome during the hospitalization. Few events occurred during hospitalization; as a consequence, the imprecise estimates are reported only in eTable 2 in Supplement 1.

We used cause-specific hazards to evaluate how cancer surgery was associated with the rate of venous thromboembolism after discharge from the hospital, censoring for competing events (ie, death from causes other than the outcome of interest).^20^ The cause-specific hazard ratios (HRs) with 95% CIs were estimated using flexible parametric models.^21^ Time since discharge was the underlying time scale and was modeled using restricted cubic splines with 4 degrees of freedom. Because the test of the null hypothesis of zero slope of the scaled Schoenfeld residuals on time^22^ indicated violation of the proportional hazards assumption for all cancer types, we allowed the effect of cancer surgery to be time-dependent by interacting it with spline terms for time. Time-dependent effects were modeled with 3 degrees of freedom. The time-dependent, cause-specific HRs were plotted over the follow-up time, and we present HRs at 30, 90, and 365 days. All modeled analyses were adjusted for the matching variables,^23^ other major surgery and comorbidities (ischemic heart disease, peripheral vascular disease, cardiac arrhythmia, cerebrovascular disease, congestive heart failure, paralysis, type 1 and 2 diabetes, chronic pulmonary disease, kidney disease, and anemia), which were a priori identified as potential confounders (eTable 3 in Supplement 1).

We also conducted sensitivity analyses. Because the Outpatient Register reached full nationwide coverage in 2001,^14^ we repeated the analyses restricted to patients who underwent cancer surgery from 2002 onwards. In addition, to exclude patients with incident subclinical pulmonary embolism, we restricted the definition of pulmonary embolism to that recorded as the main diagnosis in the Inpatient Register or the main cause of death in the Cause of Death Register. Finally, to attempt to differentiate the association of cancer from the association of surgery, where available, we used an alternative comparator population, a noncancer surgery cohort, and repeated all the above-described analyses (inclusion criteria in eTable 1 in Supplement 1).

All *P* values were obtained from 2-sided tests, and results were deemed statistically significant at *P* < .05. All analyses were conducted using Stata, versions 16.1 and 17.0 (StataCorp LP).

## Results

A total of 432 218 patients with cancer (median age, 67 years [IQR, 58-75 years]; 68.7% women) and 4 009 343 matched comparators (median age, 66 years [IQR, 57-74 years]; 69.3% women) were included in the study (Table 1; eFigure 1 in Supplement 1). Breast cancer and colorectal cancer were the largest tumor groups, constituting 37.7% and 26.3% of the cancer surgery cohort, respectively. The median length of hospitalization varied between the different cancers from 2 days (IQR, 1-4 days) for breast cancer to 17 days (IQR, 13-17 days) for bladder cancer. Of all venous thromboembolic events, 21.3% of pulmonary embolisms (1237 of 5796) and 11.6% of deep vein thromboses (1020 of 8790) occurred during the hospitalization. The baseline characteristics are presented in Table 1 for the entire population and stratified by cancer type in eTable 4 in Supplement 1.

**Table 1.** Baseline Characteristics of the Study Population

Characteristic	No. (%)	
	Cancer surgery cohort (n = 432 218)	Comparison cohort (n = 4 009 343)
Age, y		
Median (IQR)	67 (58-75)	66 (57-74)
≤49	47 622 (11.0)	469 592 (11.7)
50-59	76 306 (17.7)	739 371 (18.4)
60-69	130 311 (30.2)	1 223 306 (30.5)
70-79	115 531 (26.7)	1 038 191 (25.9)
≥80	62 448 (14.5)	538 883 (13.4)
Sex		
Female	297 056 (68.7)	2 779 805 (69.3)
Male	135 162 (31.3)	1 229 538 (30.7)
Type of cancer		
Bladder	8472 (2.0)	77 260 (1.9)
Breast	162 883 (37.7)	1 533 875 (38.3)
Colorectal	113 484 (26.3)	1 024 798 (25.6)
Gynecologic organ	57 654 (13.3)	541 225 (13.5)
Kidney and UTUC	20 893 (4.8)	192 773 (4.8)
Lung	13 890 (3.2)	128 943 (3.2)
Prostate	39 921 (9.2)	372 816 (9.3)
Gastroesophageal	15 021 (3.5)	137 653 (3.4)
Hospitalization duration, median (IQR), d^a^	6 (2-10)	NA
Other major surgery (≤1 y before index date)	20 227 (6.8)	115 620 (2.9)
Comorbidities (≤1 y before index date)		
Ischemic heart disease	14 660 (3.4)	95 292 (2.4)
Congestive heart failure	7685 (1.8)	54 590 (1.4)
Cardiac arrhythmia	15 641 (3.6)	94 909 (2.4)
Valvular disease	3374 (0.8)	19 666 (0.5)
Peripheral vascular disease	3317 (0.8)	21 095 (0.5)
Hypertension	31 689 (7.3)	141 891 (3.5)
Cerebrovascular disease	6682 (1.6)	57 789 (1.4)
Paralysis (paraplegia and hemiplegia)	590 (0.1)	4545 (0.1)
Chronic pulmonary disease	9641 (2.2)	52 435 (1.3)
Type 1 and 2 diabetes	16 277 (3.8)	96 176 (2.4)
Kidney disease	2242 (0.5)	14 907 (0.4)
Anemia	10 196 (2.4)	9881 (0.3)
Outcomes (≤1 y after index date)		
Pulmonary embolism	5796 (1.3)	7959 (0.2)
During index hospitalization	1237 (0.3)	205 (0.005)
Deep vein thrombosis	8790 (2.0)	12 100 (0.3)
During index hospitalization	1020 (0.2)	341 (0.009)
All-cause mortality	39 560 (9.2)	91 202 (2.3)
Death with pulmonary embolism^b^	1228 (0.3)	2388 (0.1)

Abbreviations: NA, not applicable; UTUC, upper urinary tract urothelial cancer.

^a^ When the date of discharge from the hospital was the same as the date of admission to the hospital, hospitalization duration equals 0.

^b^ Defined as pulmonary embolism registered in the Cause of Death Register as an underlying diagnosis or 1 of the first 3 contributing diagnoses.

### Absolute Risk of Pulmonary Embolism and Deep Vein Thrombosis

The crude ARs and ARDs of pulmonary embolism and deep vein thrombosis at 30, 90, and 365 days are presented in Table 2. The 1-year AR of pulmonary embolism and deep vein thrombosis was lowest for prostate cancer and highest for bladder cancer. The 1-year ARDs of pulmonary embolism were as follows: for bladder cancer, 2.69 percentage points (95% CI, 2.33-3.05 percentage points); for breast cancer, 0.59 percentage points (95% CI 0.55-0.63 percentage points); for colorectal cancer, 1.57 percentage points (95% CI, 1.50-1.65 percentage points); for gynecologic organ cancer, 1.32 percentage points (95% CI, 1.22-1.41 percentage points); for kidney and upper urinary tract cancer, 1.38 percentage points (95% CI, 1.21-1.55 percentage points); for lung cancer, 2.61 percentage points (95% CI, 2.34-2.89 percentage points); for gastroesophageal cancer, 2.13 percentage points (95% CI, 1.89-2.38 percentage points); and for prostate cancer, 0.57 percentage points (95% CI, 0.49-0.66 percentage points). The 1-year ARDs of deep vein thrombosis were as follows: for bladder cancer, 4.67 percentage points (95% CI, 4.21-5.14 percentage points); for breast cancer, 1.36 percentage points (95% CI 1.30-1.42 percentage points); for colorectal cancer, 2.15 percentage points (95% CI, 2.06-2.24 percentage points); for gynecologic organ cancer, 2.02 percentage points (95% CI, 1.89-2.14 percentage points); for kidney and upper urinary tract cancer, 2.14 percentage points (95% CI, 1.92-2.35 percentage points); for lung cancer, 1.40 percentage points (95% CI, 1.19-1.62 percentage points); for gastroesophageal cancer, 2.19 percentage points (95% CI, 1.94-2.45 percentage points); and for prostate cancer, 0.75 percentage points (95% CI, 0.65-0.85 percentage points).

**Table 2.** Crude Absolute Risk of Pulmonary Embolism and Deep Vein Thrombosis 30, 90, and 365 Days From Index Date

Outcome	Type of cancer							
	Bladder	Breast	Colorectal	Gynecologic organ	Kidney and UTUC	Lung	Prostate	Gastroesophogeal
**30 d**								
Pulmonary embolism								
AR in cancer cohort, %	1.13	0.10	0.63	0.47	0.70	0.65	0.42	0.98
AR in comparison cohort, %	0.02	0.01	0.02	0.02	0.01	0.01	0.01	0.02
ARD (95% CI), percentage points	1.10 (0.86-1.34)	0.09 (0.07-0.10)	0.61 (0.56-0.66)	0.45 (0.39-0.51)	0.69 (0.57-0.80)	0.64 (0.51-0.78)	0.40 (0.34-0.46)	0.96 (0.78-1.13)
Deep vein thrombosis								
AR in cancer cohort, %	0.85	0.16	0.55	0.52	1.35	0.31	0.50	0.73
AR in comparison cohort, %	0.03	0.02	0.03	0.02	0.02	0.02	0.03	0.02
ARD (95% CI), percentage points	0.81 (0.60-1.02)	0.14 (0.12-0.16)	0.52 (0.47-0.56)	0.50 (0.44-0.56)	1.33 (1.17-1.49)	0.29 (0.20-0.38)	0.47 (0.40-0.54)	0.71 (0.56-0.86)
Patients excluded, %^a^	12.0	0.1	5.6	1.3	2.5	1.0	0.1	15.1
**90 d**								
Pulmonary embolism								
AR in cancer cohort, %	1.84	0.23	1.02	0.85	1.02	1.18	0.57	1.56
AR in comparison cohort, %	0.06	0.04	0.06	0.04	0.05	0.04	0.04	0.06
ARD (95% CI), percentage points	1.77 (1.49-2.06)	0.20 (0.17-0.22)	0.96 (0.90-1.02)	0.81 (0.73-0.88)	0.97 (0.84-1.11)	1.14 (0.96-1.32)	0.52 (0.45-0.60)	1.50 (1.30-1.70)
Deep vein thrombosis								
AR in cancer cohort, %	2.44	0.64	1.19	1.07	1.72	0.76	0.75	1.35
AR in comparison cohort, %	0.08	0.06	0.10	0.07	0.07	0.07	0.08	0.08
ARD (95% CI), percentage points	2.36 (2.03-2.69)	0.58 (0.54-0.62)	1.10 (1.03-1.16)	1.00 (0.92-1.08)	1.65 (1.47-1.82)	0.69 (0.54-0.84)	0.67 (0.59-0.76)	1.27 (1.09-1.46)
Patients excluded, %^a^	0.4	0.01	0.2	0.03	0.1	0.01	0	0.7
**365 d**								
Pulmonary embolism								
AR in cancer cohort, %	2.92	0.75	1.84	1.49	1.58	2.78	0.75	2.38
AR in comparison cohort, %	0.23	0.16	0.27	0.18	0.20	0.16	0.17	0.25
ARD (95% CI), percentage points	2.69 (2.33-3.05)	0.59 (0.55-0.63)	1.57 (1.50-1.65)	1.32 (1.22-1.41)	1.38 (1.21-1.55)	2.61 (2.34-2.89)	0.57 (0.49-0.66)	2.13 (1.89-2.38)
Deep vein thrombosis								
AR in cancer cohort, %	5.00	1.62	2.52	2.28	2.46	1.68	1.07	2.52
AR in comparison cohort, %	0.33	0.26	0.37	0.26	0.32	0.27	0.32	0.32
ARD (95% CI), percentage points	4.67 (4.21-5.14)	1.36 (1.30-1.42)	2.15 (2.06-2.24)	2.02 (1.89-2.14)	2.14 (1.92-2.35)	1.40 (1.19-1.62)	0.75 (0.65-0.85)	2.19 (1.94-2.45)
Patients excluded, %^a^	0	0	0	0	0	0	0	0

Abbreviations: AR, absolute risk; ARD, absolute risk difference; UTUC, upper urinary tract urothelial cancer.

^a^ Patients who were still hospitalized for the index hospitalization at the time of analysis.

### Pulmonary Embolism and Deep Vein Thrombosis After Discharge

The temporal pattern of cause-specific HRs for pulmonary embolism after discharge varied between different cancer types (Figure, A and B, and Table 3). In the early postoperative phase, patients in the cancer surgery cohort had a 10-fold to 30-fold increase in the cause-specific hazard of pulmonary embolism compared with the matched comparators for all cancers except breast cancer (colorectal cancer: HR, 9.18 [95% CI, 8.03-10.50]; lung cancer: HR, 25.66 [95% CI, 17.41-37.84]; breast cancer: HR, 5.18 [95% CI, 4.45-6.05]). After the initial peak, the HRs decreased until they plateaued, without ever reaching the level of matched comparison subjects. The 1-year HR was highest for lung cancer (HR 8.89; 95% CI, 5.97-13.22). Only for prostate cancer, the cause-specific HR approached that of the matched comparators at approximately 90 days (Figure, A) and remained the same until the end of follow-up at 1 year (HR, 1.22; 95% CI, 0.82-1.83). Unlike the other cancers, there was no clear postoperative peak of rate of pulmonary embolism among patients with breast cancer (Figure, B).

**Figure.**
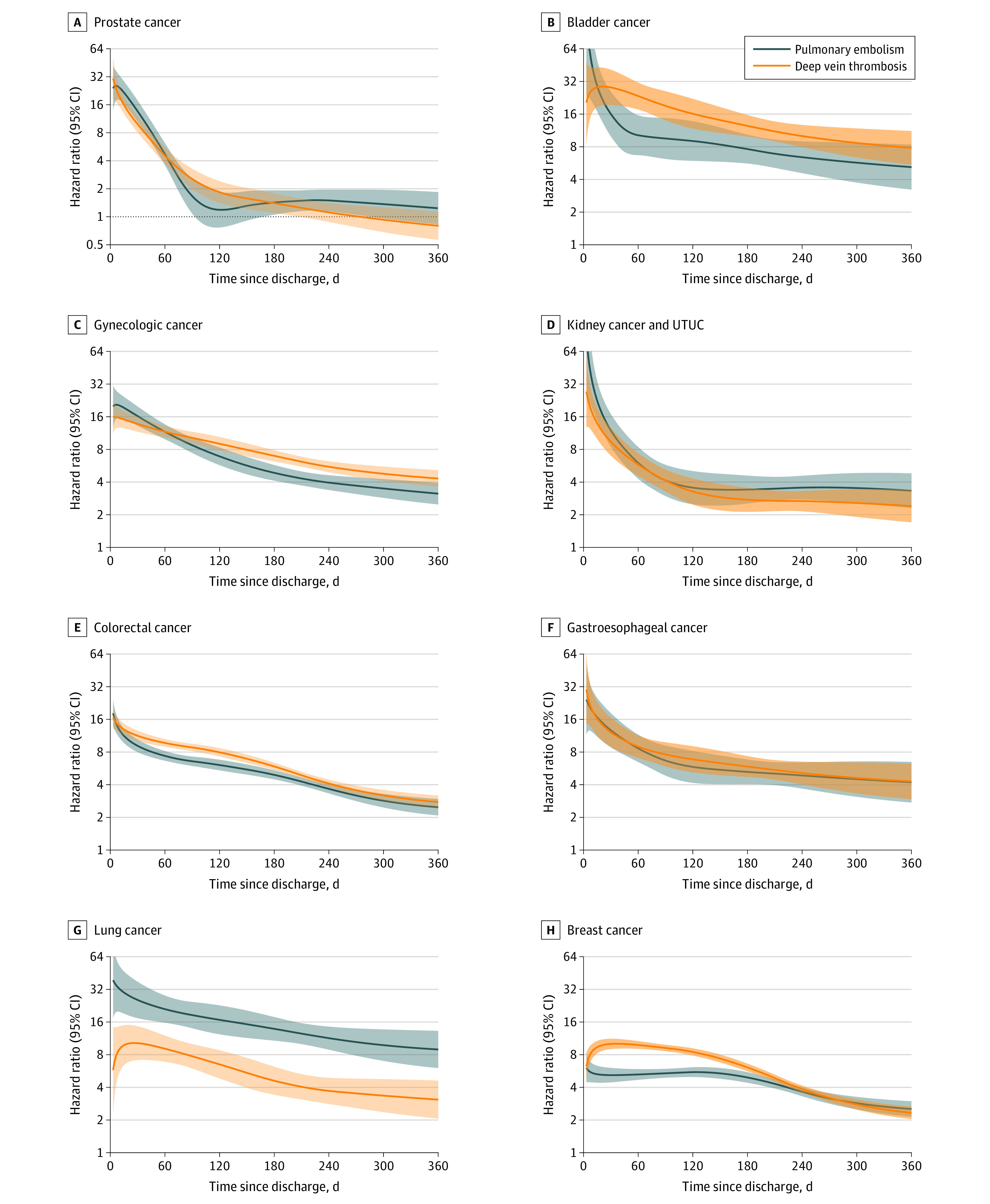
Risk of Pulmonary Embolism and Deep Vein Thrombosis After Discharge From the Hospital for Cancer Surgery vs Comparison Cohort UTUC indicates upper urinary tract urothelial cancer.

**Table 3.** Risk of Pulmonary Embolism After Discharge From the Hospital for Cancer Surgery vs Comparison Cohort^a^

Type of cancer	Cancer surgery vs comparison cohort			Female sex^b^^,^^c^	Age, y^c^				
	At 30 d	At 90 d	At 365 d		≤49	50-59	60-69	70-79	≥80
Bladder	16.33 (10.18-26.22)	9.55 (6.19-14.73)	5.16 (3.19-8.35)	0.84 (0.65-1.10)	0.40 (0.16-0.99)	0.64 (0.41-0.97)	1 [Reference]	1.28 (1.01-1.64)	1.38 (0.93-2.04)
Breast	5.18 (4.45-6.05)	5.39 (4.90-5.92)	2.51 (2.13-2.96)	1.37 (0.87-2.14)	0.32 (0.27-0.37)	0.43 (0.37-0.48)	1 [Reference]	1.83 (1.68-1.99)	2.69 (2.46-2.94)
Colorectal	9.18 (8.03-10.50)	6.59 (5.97-7.28)	2.46 (2.07-2.94)	0.97 (0.91-1.03)	0.52 (0.42-0.66)	0.61 (0.53-0.71)	1 [Reference]	1.30 (1.19-1.41)	1.69 (1.55-1.85)
Gynecologic organ	16.31 (13.50-20.00)	8.78 (7.46-10.34)	3.11 (2.47-3.91)	NA	0.25 (0.19-0.33)	0.54 (0.45-0.64)	1 [Reference]	1.66 (1.46-1.88)	2.02 (1.75-2.34)
Kidney and UTUC	12.19 (8.60-17.28)	4.17 (3.00-5.80)	3.31 (2.29-4.80)	0.91 (0.77-1.07)	0.35 (0.22-0.56)	0.46 (0.33-0.65)	1 [Reference]	1.29 (1.06-1.57)	1.92 (1.51-2.43)
Lung	25.66 (17.41-37.84)	18.56 (14.06-24.51)	8.89 (5.97-13.22)	0.91 (0.77-1.07)	0.43 (0.26-0.70)	0.8 (0.61-1.05)	1 [Reference]	1.18 (0.97-1.43)	1.81 (1.27-2.57)
Prostate	13.90 (10.67-18.11)	1.70 (1.10-2.60)	1.22 (0.82-1.83)	NA	0.00 (0.00)	0.62 (0.51-0.76)	1 [Reference]	1.38 (1.18-1.62)	0.99 (0.14-7.08)
Gastroesophageal	12.83 (8.91-18.47)	6.71 (4.89-9.22)	4.20 (2.72-6.48)	0.87 (0.72-1.05)	0.80 (0.49-1.30)	0.65 (0.45-0.96)	1 [Reference]	1.62 (1.28-2.06)	2.44 (1.88-3.16)

Abbreviations: NA, not applicable; UTUC, upper urinary tract urothelial cancer.

^a^ Data are presented as hazard ratio (95% CI).

^b^ Male sex as reference.

^c^ Time-fixed hazard ratios.

The temporal patterns of cause-specific HRs for deep vein thrombosis after discharge are presented in eTable 5 in Supplement 1. Although the association of cancer surgery and deep vein thrombosis mimics that for pulmonary embolism, the HRs for deep vein thrombosis were higher but with overlapping 95% CIs, except for lung cancer surgery (Figure, A and B). For breast cancer surgery, the HR of deep vein thrombosis was higher at 30 and 90 days after-surgery than for pulmonary embolism (Figure, B, and Table 3; eTable 5 in Supplement 1).

### Age and Sex

Age was independently associated with the rate of postdischarge venous thromboembolism, with an increasing cause-specific HR with increasing age (eg, for colorectal cancer the HR increased from 0.52 [95% CI, 0.42-0.66] for patients aged ≤49 years to 1.30 [95% CI, 1.19-1.41] for those aged 70-79 years, where the reference is age 60-69 years) (Table 3; eTable 5 in Supplement 1). There was no clear association between sex and a postdischarge venous thromboembolic event.

### Sensitivity Analysis

In the analyses restricted to patients who underwent surgery after 2001 (eTables 6-8, eFigure 2 in Supplement 1) and confining the outcome to main diagnoses only, the results remained virtually unchanged (eTables 9-11, eFigure 3 in Supplement 1). When we compared the cancer surgery cohort with the noncancer surgery cohort, we observed either minimal variation of cause-specific HRs over time (prostate and kidney cancers), or even lower HRs during the first 60 days after discharge, which then increased and plateaued at a level comparable with the main analysis (gynecologic and colorectal cancers), both indicating that the early postoperative increase in HR in the main analysis is likely due to surgery (eFigure 4 in Supplement 1).

## Discussion

In this large nationwide cohort of patients who underwent cancer surgery, we found an increased 1-year cumulative risk of venous thromboembolism compared with a matched comparison cohort. The risk difference varied from 0.57 to 4.67 percentage points depending on the type of cancer surgery and type of thromboembolic event. The cause-specific HR was highest just after surgery, after which it subsided and plateaued at approximately 90 to 120 days, when the surgical trauma likely waned and only the baseline risk due to cancer remained. The adjusted HRs varied between the different cancer surgical procedures.

Venous thromboembolism is a rare unintended outcome after surgery that is particularly suitable for research using clinical data. We were able to use the Swedish health care and population registers, which are known for their high coverage, completeness, and accuracy,^13^ to assemble a large cohort of patients who underwent cancer surgery and estimate risks in comparison with a matched population with high statistical precision.

Previous studies have reported similar absolute risks for venous thromboembolic events among patients undergoing cancer surgery, although without a matched background population to assess the excess risk associated with the cancer surgery. Mallick et al^24^ found that venous thromboembolic event-related readmissions in the US Nationwide Readmissions Database continued to increase well beyond 30 days. In their study they found a 1.7% rate of 180-day readmission for venous thromboembolism. Jarvis et al^25^ used the same database and, similar to our findings, reported different risks after different cancer surgical procedures. In our study, a nonnegligible proportion of patients were still hospitalized at 30 days. Because long hospitalization is likely associated with a higher risk of thromboembolism, our 30-day absolute risks of pulmonary embolism are presumably underestimating the true risk.

Similar occurrences of venous thromboembolic events were suggested by a meta-analysis, primarily concentrating on outcomes at 4 weeks and 3 months after surgery, with highest risk for outcomes during the first 4 weeks and an elevated risk throughout the study period.^26^ It is, however, unclear from the protocol if timing of venous thromboembolic events during hospitalization was known. The study did not examine only cancer surgery, and pooled estimates from the meta-analysis hide the differences between different types of cancer surgery, as presented in our study. Their findings confirm our data suggesting that the elevated incidence of venous thromboembolic events persists long after the surgery.

Although we were unable to disentangle the association of the surgery with venous thromboembolism from the association of the cancer with venous thromboembolism, the variation in the patterns of occurrence of venous thromboembolism between the different cancer types allows us to hypothesize on the association of the surgical procedure with venous thromboembolism. For example, breast cancer treatment entails a limited surgical trauma frequently followed by systemic chemotherapy and radiotherapy. The rate of thromboembolism after such a treatment regimen may be reflected by the lack of a transient postoperative peak followed by a longstanding increased rate during the first year after surgery. In contrast, prostate cancer surgery is a more traumatic procedure, especially if combined with a lymph node dissection, but it is rarely associated with any postoperative adjuvant treatment, and is performed in an otherwise healthy population. In this case, the rate of thromboembolism after surgery may be mirrored by the high surgery-associated transient peak during the first 90 days, which thereafter, due to the lack of cancer specific risks, coincides with the rate of the background population. This speculation is further corroborated by the comparison with the noncancer surgery cohort in which the transitory postsurgery spike in thromboembolic event rate is likely due to surgical trauma.

For most cancers, there is a noticeable transient postoperative increase in the rate of venous thromboembolic events lasting around 90 to 120 days followed by a continuous, more stable but lower increased rate that may be associated with either advanced disease or thrombogenic effects of adjuvant treatments. Pelvic lymph node dissections have been associated with a significant increase in risk of thromboembolism,^27,28^ which may explain why prostate, bladder, and gynecologic cancer surgery have higher peaks in rates of venous thromboembolic events than surgery for colorectal cancer, which is less frequently combined with a separate extensive lymph node dissection. In more aggressive cancers, such as bladder and lung cancer, we see a higher 1-year cumulative incidence of venous thromboembolic events. This higher incidence is probably associated with the high risk of advanced disease and progression to poorer prognosis in combination with extensive surgical procedures, leading to higher rates of postoperative chemotherapy, metastases, and death. Thus, among patients with more aggressive cancers, there may also be a higher prevalence of systemic treatments with potential thrombogenic effects.

The marked variation in the occurrence patterns of postoperative venous thromboembolic events indicates a need for a more tailored approach to prophylaxis. Although venous thromboembolism is most common in the period covered by standard prophylactic regimens, the transient peak of the rate of venous events is, in general, of longer duration than such regimens, including extended prophylaxes. Our observational data alone cannot, however, bring about a change in clinical practice, and future studies should evaluate individualized prophylactic regimens by taking the surgical trauma, disease severity, and exposure to systemic chemotherapy into account, to avoid both overtreatment and undertreatment in the prevention of venous thromboembolic events.

### Limitations

This study has some limitations. One major limitation is that we lack information on treatments besides surgery that may be associated with increased risks of venous thromboembolism.^29,30,31^ Furthermore, changes in clinical practices and diagnostics over time could affect both the occurrence and detection of the outcomes. The change in surgical practices to more minimally invasive techniques, wider use of thromboprophylaxis, introduction of extended thromboprophylaxis during the study period, increased availability of better diagnostics, and a shift toward operating on older patients with more comorbidities over time could all be associated with the outcome. The lack of information on thromboprophylaxis in the surgery cohort could also be seen as another limitation. However, what we tried to estimate in this study corresponds to a total association of cancer surgery with outcomes. Thromboprophylaxis is a mediator in this setting, and adjusting for it would, at best, lead to a different estimate or, at worst, introduce bias in the presence of unmeasured confounding of mediator outcome association. However, because we know that many patients undergoing cancer surgery received prophylaxis, and the control population likely did not, we can speculate that the true untreated risk among the cancer surgery cohort should not be lower than presented. Finally, we used a cause-specific hazards approach to account for competing events. When censoring due to the competing events is informative, the association with the rate of venous thromboembolism may not translate to the association with the cause-specific cumulative incidence. However, for most cancers included in this study, competing events were rare during follow-up, especially in the first 120 days, when the transient association of cancer surgery was observed.

## Conclusions

In this cohort study of postoperative venous thromboembolic events among patients who underwent cancer surgery, we found that the elevated relative risk of deep vein thrombosis and pulmonary embolism extends beyond an in-hospital or 28-day extended thromboprophylaxis. The 1-year postoperative risks of pulmonary embolism or deep vein thrombosis were different for different cancers, ranging from 0.57 to 4.67 percentage points, which should be considered in future prophylactic regimens. The results highlight the need for individualized venous thromboembolism risk evaluation and prophylaxis regimens for patients undergoing surgery for different cancers.

## Notes

Supplement 1. **eTable 1.** Inclusion Diagnostic and Procedural Codes **eFigure 1.** Flow Chart of the Patient Selection **eTable 2.** The Odds Ratios and 95% Confidence Intervals of Pulmonary Embolism and Deep Vein Thrombosis During Hospitalization for Cancer Surgery vs Comparison Cohort, by Cancer Type **eTable 3.** Sources and Definition of Confounders **eTable 4.** Patient Demographics for Separate Tumor Forms **eTable 5.** The Hazard Ratios and the 95% Confidence Intervals of Deep Vein Thrombosis After the Discharge From the Hospital for Cancer Surgery vs Comparison Cohort, by Cancer Type **eTable 6.** Sensitivity Analysis Restricted to Subjects Operated From 2002 Onwards: Crude Absolute Risk of Pulmonary Embolism and Deep Vein Thrombosis 30, 90 and 365 Days From Index Date, by Cancer Type **eTable 7.** Sensitivity Analysis Restricted to Subjects Operated From 2002 Onwards: Adjusted Odds Ratios and 95% Confidence Intervals of Pulmonary Embolism and Deep Vein Thrombosis During Hospitalization for Cancer Surgery vs Comparison Cohort, by Cancer Type **eTable 8.** Sensitivity Analysis Restricted to Subjects Operated From 2002 Onwards: Hazard Ratios and the 95% Confidence Intervals of Pulmonary Embolism and Deep Vein Thrombosis After the Discharge From the Hospital for Cancer Surgery vs Comparison Cohort, by Cancer Type **eFigure 2.** The Plot of Hazard Ratios and Corresponding 95% Confidence Intervals of Pulmonary Embolism and Deep Vein Thrombosis After the Discharge From the Hospital for Cancer Surgery vs Comparison Cohort, by Cancer Type **eTable 9.** Sensitivity Analysis in Which Pulmonary Embolism Was Defined as a Main Inpatient Diagnosis or Underlying Cause of Death: Crude Absolute Risk of Pulmonary Embolism 30, 90 and 365 Days From Index Date, by Cancer Type **eTable 10.** Sensitivity Analysis in Which Pulmonary Embolism Was Defined as a Main Inpatient Diagnosis or Underlying Cause of Death: Odds Ratios and the 95% Confidence Intervals of Pulmonary Embolism During Hospitalization for Cancer Surgery vs Comparison Cohort, by Cancer Type **eTable 11.** Sensitivity Analysis in Which Pulmonary Embolism Was Defined as a Main Inpatient Diagnosis or Underlying Cause of Death: Hazard Ratios and the 95% Confidence Intervals of Pulmonary Embolism After the Discharge From the Hospital for Cancer Surgery vs Comparison Cohort, by Cancer Type **eFigure 3.** Sensitivity Analysis in Which Pulmonary Embolism Was Defined as a Main Inpatient Diagnosis or Underlying Cause of Death: the Plot of Hazard Ratios and Corresponding 95% Confidence Intervals of Pulmonary Embolism After the Discharge From the Hospital for Cancer Surgery vs Comparison Cohort, by Cancer Type **eFigure 4.** Sensitivity Analysis Comparing Cancer Surgery to Benign Surgery Patients: the Plot of Hazard Ratios and Corresponding 95% Confidence Intervals for Pulmonary Embolism and Deep Vein Thrombosis After the Discharge From the Hospital, by Cancer Type

Supplement 2.
Data Sharing Statement
