# Supplementary material for: Risk of Venous Thromboembolic Events After Surgery for Cancer
Source: JAMA Netw Open. 2024 Feb 2;7(2):e2354352. doi: 10.1001/jamanetworkopen.2023.54352 (PMC10837742; doi:10.1001/jamanetworkopen.2023.54352)
Supplement: Supplement 1. — eTable 1. Inclusion Diagnostic and Procedural Codes eFigure 1. Flow Chart of the Patient Selection eTable 2. The Odds Ratios and 95% Confidence Intervals of Pulmonary Embolism and Deep Vein Thrombosis During Hospitalization for Cancer Surgery vs Comparison Cohort, by Cancer Type eTable 3. Sources and Definition of Confounders eTable 4. Patient Demographics for Separate Tumor Forms eTable 5. The Hazard Ratios and the 95% Confidence Intervals of Deep Vein Thrombosis After the Discharge From the Hospital for Cancer Surgery vs Comparison Cohort, by Cancer Type eTable 6. Sensitivity Analysis Restricted to Subjects Operated From 2002 Onwards: Crude Absolute Risk of Pulmonary Embolism and Deep Vein Thrombosis 30, 90 and 365 Days From Index Date, by Cancer Type eTable 7. Sensitivity Analysis Restricted to Subjects Operated From 2002 Onwards: Adjusted Odds Ratios and 95% Confidence Intervals of Pulmonary Embolism and Deep Vein Thrombosis During Hospitalization for Cancer Surgery vs Comparison Cohort, by Cancer Type eTable 8. Sensitivity Analysis Restricted to Subjects Operated From 2002 Onwards: Hazard Ratios and the 95% Confidence Intervals of Pulmonary Embolism and Deep Vein Thrombosis After the Discharge From the Hospital for Cancer Surgery vs Comparison Cohort, by Cancer Type eFigure 2. The Plot of Hazard Ratios and Corresponding 95% Confidence Intervals of Pulmonary Embolism and Deep Vein Thrombosis After the Discharge From the Hospital for Cancer Surgery vs Comparison Cohort, by Cancer Type eTable 9. Sensitivity Analysis in Which Pulmonary Embolism Was Defined as a Main Inpatient Diagnosis or Underlying Cause of Death: Crude Absolute Risk of Pulmonary Embolism 30, 90 and 365 Days From Index Date, by Cancer Type eTable 10. Sensitivity Analysis in Which Pulmonary Embolism Was Defined as a Main Inpatient Diagnosis or Underlying Cause of Death: Odds Ratios and the 95% Confidence Intervals of Pulmonary Embolism During Hospitalization for Cancer Surgery vs Comparison Cohort, [file jamanetwopen-e2354352-s001.pdf]

Supplementary Online Content

Björklund J, Rautiola J, Zelic R, et al. Risk of venous thromboembolic events after surgery for cancer: a nationwide population-based cohort study. *JAMA Netw Open.* 2024;7(2):e2354352. doi:10.1001/jamanetworkopen.2023.54352

eTable 1. Inclusion Diagnostic and Procedural Codes
eFigure 1. Flow Chart of the Patient Selection
eTable 2. The Odds Ratios and 95% Confidence Intervals of Pulmonary Embolism and Deep Vein Thrombosis During Hospitalization for Cancer Surgery vs Comparison Cohort, by Cancer Type
eTable 3. Sources and Definition of Confounders
eTable 4. Patient Demographics for Separate Tumor Forms
eTable 5. The Hazard Ratios and the 95% Confidence Intervals of Deep Vein Thrombosis After the Discharge From the Hospital for Cancer Surgery vs Comparison Cohort, by Cancer Type
eTable 6. Sensitivity Analysis Restricted to Subjects Operated From 2002 Onwards: Crude Absolute Risk of Pulmonary Embolism and Deep Vein Thrombosis 30, 90 and 365 Days From Index Date, by Cancer Type
eTable 7. Sensitivity Analysis Restricted to Subjects Operated From 2002 Onwards: Adjusted Odds Ratios and 95% Confidence Intervals of Pulmonary Embolism and Deep Vein Thrombosis During Hospitalization for Cancer Surgery vs Comparison Cohort, by Cancer Type
eTable 8. Sensitivity Analysis Restricted to Subjects Operated From 2002 Onwards: Hazard Ratios and the 95% Confidence Intervals of Pulmonary Embolism and Deep Vein Thrombosis After the Discharge From the Hospital for Cancer Surgery vs Comparison Cohort, by Cancer Type
eFigure 2. The Plot of Hazard Ratios and Corresponding 95% Confidence Intervals of Pulmonary Embolism and Deep Vein Thrombosis After the Discharge From the Hospital for Cancer Surgery vs Comparison Cohort, by Cancer Type
eTable 9. Sensitivity Analysis in Which Pulmonary Embolism Was Defined as a Main Inpatient Diagnosis or Underlying Cause of Death: Crude Absolute Risk of Pulmonary Embolism 30, 90 and 365 Days From Index Date, by Cancer Type
eTable 10. Sensitivity Analysis in Which Pulmonary Embolism Was Defined as a Main Inpatient Diagnosis or Underlying Cause of Death: Odds Ratios and the 95% Confidence Intervals of Pulmonary Embolism During Hospitalization for Cancer Surgery vs Comparison Cohort, by Cancer Type
eTable 11. Sensitivity Analysis in Which Pulmonary Embolism Was Defined as a Main Inpatient Diagnosis or Underlying Cause of Death: Hazard Ratios and the 95% Confidence Intervals of Pulmonary Embolism After the Discharge From the Hospital for Cancer Surgery vs Comparison Cohort, by Cancer Type
eFigure 3. Sensitivity Analysis in Which Pulmonary Embolism Was Defined as a Main Inpatient Diagnosis or Underlying Cause of Death: the Plot of Hazard Ratios and Corresponding 95% Confidence Intervals of Pulmonary Embolism After the Discharge From the Hospital for Cancer Surgery vs Comparison Cohort, by Cancer Type
eFigure 4. Sensitivity Analysis Comparing Cancer Surgery to Benign Surgery Patients: the Plot of Hazard Ratios and Corresponding 95% Confidence Intervals for Pulmonary Embolism and Deep Vein Thrombosis After the Discharge From the Hospital, by Cancer Type

This supplementary material has been provided by the authors to give readers additional information about their work.

© 2024 Björklund J et al. *JAMA Network Open.*

eTable 1. Inclusion Diagnostic and Procedural Codes

Cancer type	ICD codes[1]		Procedural codes	
	ICD-9	ICD-10	K06	KVÅ
Cancer surgery cohort				
Bladder	188x	C67x	6318, 6320–28, 6331	KCC00, KCC10, KCC20, KCC30, KCC96
Breast	174x, 175	C50x	3801–02, 3810-14, 3816-17, 3820-21, 3830-32, 3840, 3890-91	HAB00, HAB20, HAB30, HAB40, HAB99, HAC10, HAC15, HAC20, HAC22, HAC25, HAC30, HAC99
Colorectal	153x, 154x	C18x, C19x, C20x	4640-44, 4648-54, 4810, 4820-23, 4828-29	JFA83, JFB20-21, JFB30-31, JFB33-34, JFB40-41, JFB43-44, JFB46-47, JFB50-51, JFB53-54, JFB60-61, JFB63-64, JFB96-97, JGB00-01, JGB03-04, JGB10-11, JGB20, JGB30-31, JGB33-34, JGB36, JGB40, JGB60-61, JGB96-97, JFH00-01, JFH10-11, JFH20, JFH30-31, JFH33, JFH40, JFH96
Gallbladder	156x	C23, C24x	5310-13, 5319, 5350-53, 5356-57, 5359, 5398-99	JKA20-21, JKA96-97, JKC00-01, JKC10, JKC20, JKC30, JKC40, JKC50, JKC96-97, JKW96-98
Gastroesophageal	150x, 151x	C15x, C16x	2820-22, 2829, 4411-26, 4429-30, 4432, 4434-35, 4439	JCC00, JCC10-12, JCC20, JCC30, JCC96-97, JDC00, JDC10-11, JDC20, JDC30, JDC40, JDC96-97, JDD00-01, JDD96
Gynecological	179x, 180x, 182x, 183x, 184x	C53x, C54x, C55, C56, C57x	7010-12,7020-23, 7030-33, 7120-21, 7123, 7210-11, 7214-18, 7220-23, 7228-29, 7240, 7249-52, 7259-63, 7269, 7310-11	LAD00-01, LAE10-11, LAE20-21, LAF00-01, LAF10-11, LAF20, LAF30, LDB00, LBE00-01, LBE03, LCB97-98, LCC00-01, LCC05, LCC10-11, LCC20, LCC96-97, LCD00-01, LCD04, LCD10-11, LCD30-31, LCD40, LCD96-97, LCE00, LCE10, LCE20, LCE96, LCF00-01, LCF96-97, LDC10, LDC96
Kidney and UTUC	189x	C64, C65, C66	6020, 6030, 6040-43	KAC00-01, KAC20-21, KAD00-01, KAD10, KAD40, KAD51-52, KAD56, KAD60, KAD96-98, KBC00, KBD00, KBD22
Lung	162x	C34x	3520, 3530, 3532, 3534-35, 3539-43	GDB00-01, GDB10-11, GDB20-21, GDB96-97, GDC00-01, GDC10-11, GDC13, GDC20, GDC23, GDC26, GDC96-97, GDD00-01, GDD10-11, GDD20, GDD23, GDD26, GDD96-97
Pancreas	157x	C25x	5510-17, 5519	JLC00, JLC10-11, JLC20, JLC30, JLC40, JLC50, JLC96
Prostate	185	C61	6611, 6631, 6633, 6639	KEC00-01, KEC10, KEC20
Testicular	186x	C62x	6740-42, 6751-52, 6120	KFC00, KFC10, KFC15, KFC96, KFD00
Benign surgery cohort				
Bladder	NA	NA	6318, 6320, 6321, 6322, 6324, 6325, 6327, 6328, 6331	KCC00, KCC10, KCC20, KCC30
Colorectal	NA	NA	4640-44, 4648-54, 4820-22, 4828	JFB20-21, JFB30-31, JFB33-34, JFB40-41, JFB43-44, JFB46-47, JFB50-51, JFB53-54, JFB60-61, JFB63-64, JFB96-97, JFH00-01, JFH10-11, JFH20, JFH30-31, JFH33, JFH40, JFH96, JGB00-01, JGB03-04,

© 2024 Björklund J et al. *JAMA Network Open.*

Cancer type	ICD codes[1]		Procedural codes	
	ICD-9	ICD-10	K06	KVÅ
Gynecological			7010-11, 7020-22, 7031-32, 7210-11, 7220-23, 7229, 7261-63	JGB10-11, JGB20, JGB30-31, JGB33-34, JGB36, JGB40, JGB50, JGB60-61, JGB96-97 LCC00-01, LCC05, LCC10-11, LCC20, LCC96-97, LCD00-01, LCD04, LCD10-11, LCD30-31, LCD40, LCD96-97, LAD00-01, LAE10-11, LAE20-21, LAF00-01, LAF10-11, LAF20, LAF30
Kidney and UTUC	NA	NA	6040, 6041, 6042, 6043	KAC01, KAC20, KAC21
Prostate	NA	NA	6610, 6630, 6632	KED00

Abbreviations: ICD, International Classification of Disease (Swedish version); K06, The national classification for operations, sixth edition (1988); KVÅ, Klassifikation av vårdåtgärder, an adapted version of NOMESCO classification of surgical procedures; NA, Not applicable
[1] ICD-9 and ICD-10 codes recorded as a main, or any of the 30 contributory diagnoses

© 2024 Björklund J et al. *JAMA Network Open.*

eFigure 1. Flow Chart of the Patient Selection

Note: When a cancer patient was excluded, we also excluded all comparison cohort comparators. Special cases are further explained in notes.

Abbreviations: DVT, Deep Vein thrombosis; PE, Pulmonary Embolism; UTUC, upper tract urothelial cancer

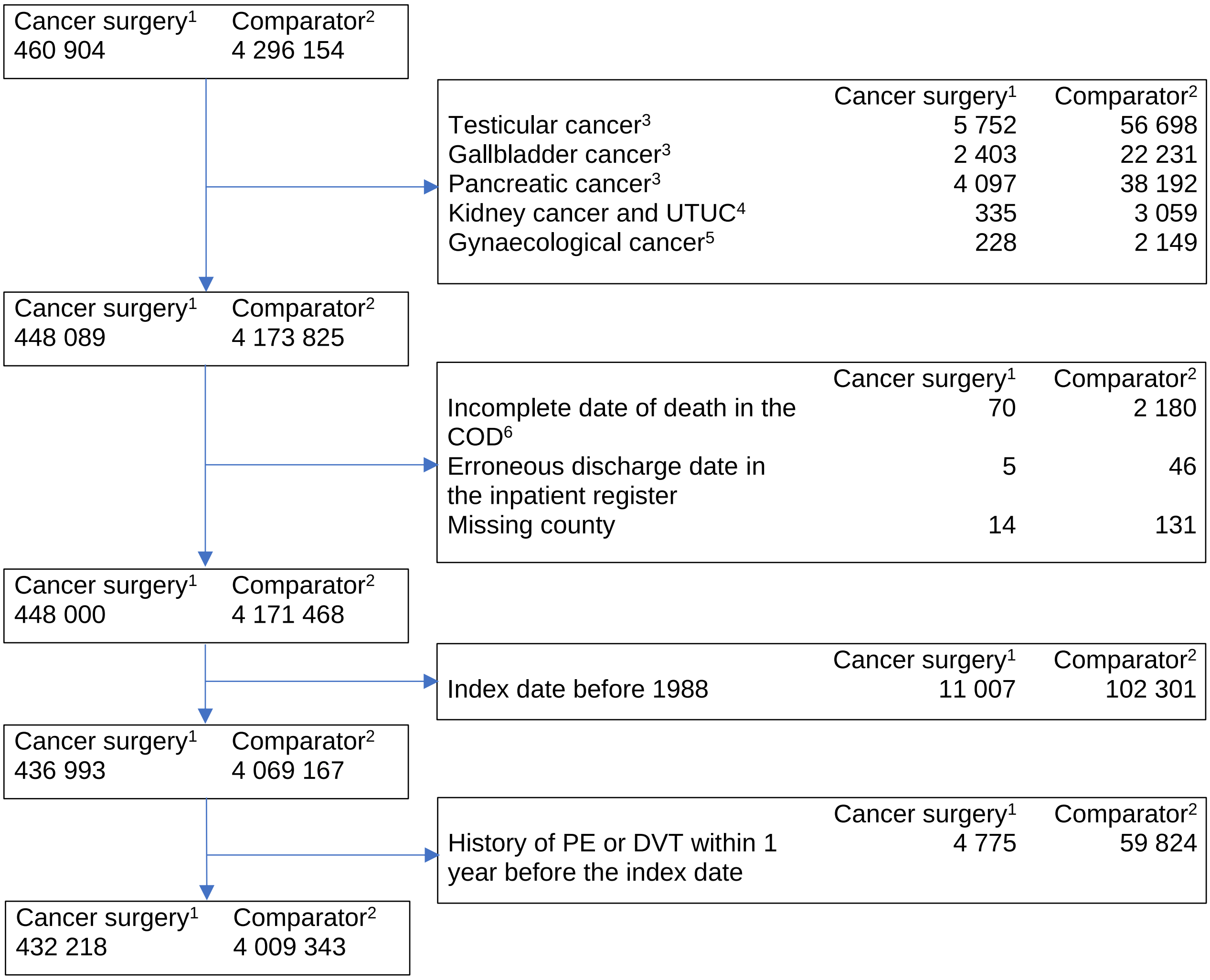

[1] All patients with selected cancers who were treated with surgery (see Table S1 for details on the inclusion criteria i.e., cancer and surgery codes).

[2] General population comparison subjects who were cancer free one year before the index date, matched on birth year, sex and county.

[3] All patients with testicular, pancreatic and gallbladder cancer were excluded due to potential issues with data quality for these cancer cohorts.

[4] Patients with kidney cancer or UTUC surgery excluded if they had surgery code KAD52, KAD56, KAD98 or KAD60 recorded without at least one of the following codes: KAC20, KAD00, KAD01, KAB00, KAB01, JAH01, KAC00, PJD41, PJD42, PJD43, PJD44, PJD45, PJD51, PJD52, PJD53, PJD54, PJD55, PJD98 or PJD99. These patients were considered not having a major surgery.

[5] Patients with gynecological cancer surgery excluded if they had surgery code LDB00 recorded without at least one of the following codes: JAH00, LAF10, LAF20, JAL30, LCD00, LCD10, PJD41, PJD42, PJD43, PJD44, PJD45, PJD51, PJD52, PJD53, PJD54, PJD55, PJD98 or PJD99. These patients were considered not having a major surgery.

[6] Incomplete date of death refers to date records having 00 as the day and/or 00 as the month of death. If a matched comparison subject had an incomplete date of death in the Cause of death register, only that subject was excluded. If a cancer surgery patient had an incomplete date of death in the Cause of death register, that subject and all matched comparison subjects were excluded. A subject who died on the day 00 had to survive at least 1 year + 1 month from the index date not to be excluded. A subject who died on the month 00 had to survive a minimum of 1 year from the end of the index year not to be excluded.

© 2024 Björklund J et al. *JAMA Network Open.*

Further selection for analysis of the outcome after the discharge

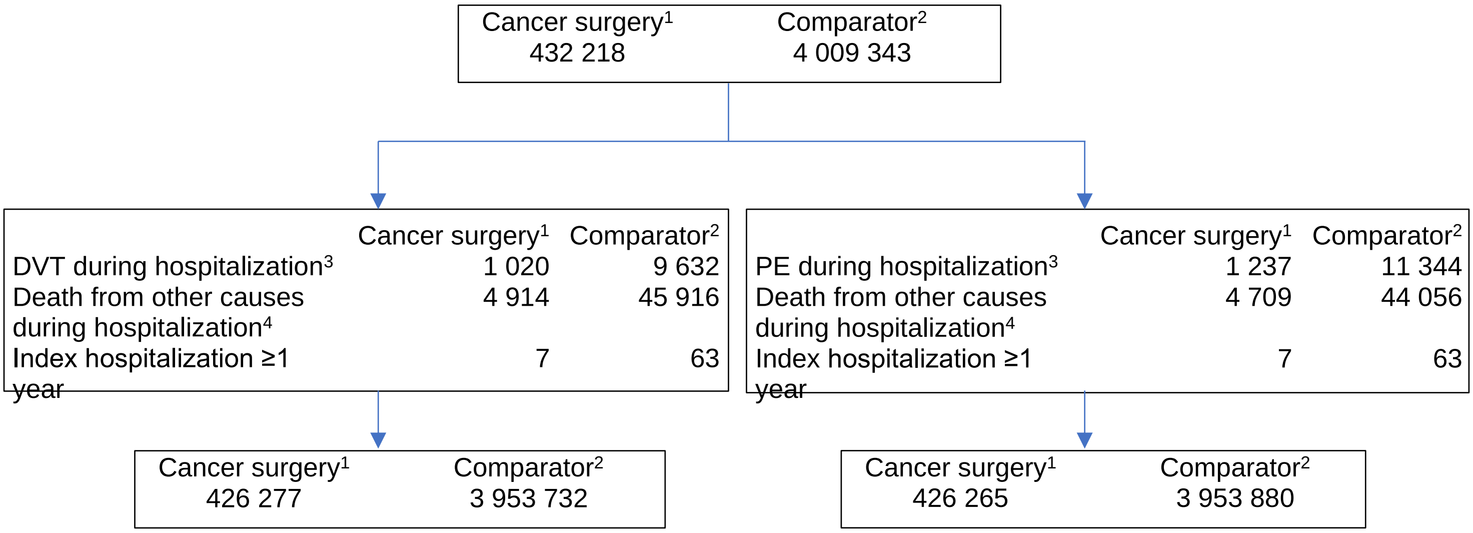

[1] All patients with selected cancers who were treated with surgery (see Table S1 for details on the inclusion criteria i.e., cancer and surgery codes).
[2] General population comparison subjects who were cancer free one year before the index date, matched on birth year, sex and county.
[3] If a matched comparison subject had an outcome during hospitalization, only that subject was excluded. If a cancer surgery patient had an outcome during hospitalization, that subject and all matched comparison subjects were excluded.
[4] If a matched comparison subject had died from causes other than outcome during hospitalization, only that subject was excluded. If a cancer surgery patient had died from causes other than outcome during hospitalization, that subject and all matched comparison subjects were excluded.

© 2024 Björklund J et al. *JAMA Network Open.*

eTable 2. The Odds Ratios and 95% Confidence Intervals of Pulmonary Embolism and Deep Vein Thrombosis During Hospitalization for Cancer Surgery vs Comparison Cohort, by Cancer Type

Cancer type	PE		DVT	
	OR	95 % CIs	OR	95 % CIs
Bladder	66.83	34.30, 130.20	34.01	19.10, 60.55
Breast	20.30	12.26, 33.63	10.71	7.14, 16.04
Colorectal	53.14	42.91, 65.81	19.14	15.94, 22.98
Gynecological	56.67	37.40, 85.87	37.10	25.52, 53.93
Kidney and UTUC	86.96	46.51, 162.59	133.67	80.25, 222.63
Lung	88.30	31.07, 250.91	18.71	8.03, 43.60
Prostate	60.28	28.47, 127.64	14.92	8.02, 27.78
Gastroesophageal	57.74	36.65, 90.99	39.00	23.18, 65.61

Abbreviations: PE, pulmonary embolism; DVT, deep vein thrombosis; OR, odds ratios; CI, Confidence interval; UTUC, upper tract urothelial cancer

[1] All models adjusted for the matching variables, other major surgery, and comorbidities (ischemic heart disease, peripheral vascular disease, cardiac arrhythmia, cerebrovascular disease, congestive heart failure, paralysis, hypertension, diabetes mellitus, chronic pulmonary disease, renal disease, and anemia)

© 2024 Björklund J et al. *JAMA Network Open.*

eTable 3. Sources and Definition of Confounders

Confounders	Source and definition	Time before index date
Comorbidities		< 1 year[2]
Ischemic heart disease	NPR; ICD-9[1]: 410-413, 414A, 414B, 414W, 414X, 416X, 423A, 429C, 429F, 429G, 429W; ICD-10[1]: I20-I25	< 1 year[2]
Peripheral Vascular Disease	NPR; ICD-9[1]: 440, 441, 443, V43E; ICD-10[1]: I70, I71, I73	< 1 year[2]
Cardiac Arrhythmia	NPR; ICD-9[1]: 426, 427; ICD-10[1]: I44, I45, I47-I49, R00	< 1 year[2]
Cerebrovascular disease	NPR; ICD-9[1]: 430-438; ICD-10[1]: G45, G46, I60-I63	< 1 year[2]
Congestive Heart Failure	NPR; ICD-9[1]: 398, 414W, 422, 425, 428, 429X; ICD-10[1]: I42, I43, I50	< 1 year[2]
Paralysis (Paraplegia and Hemiplegia, including paresis)	NPR; ICD-9[1]: 342-344; ICD-10[1]: G80- G83	< 1 year[2]
Hypertension (without and with) complications	NPR; ICD-9[1]: 401-405; ICD-10[1]: I10-I13, I15	< 1 year[2]
Diabetes mellitus	NPR; ICD-9[1]: 250; ICD-10[1]: E10-14	< 1 year[2]
Chronic Pulmonary Disease	NPR; ICD-9[1]: 490-496, 501-505; ICD-10[1]: J40-47, J60-67	< 1 year[2]
Renal Disease	NPR; ICD-9[1]: 582, 583, 585, 586, 588, V42A, V56; ICD-10[1]: N03, N05, N18-19, N25, Z49	< 1 year[2]
Any anemia	NPR; ICD-9[1]: 280, 281; ICD-10[1]: D50, D51-D53	< 1 year[2]
Other major surgeries	NPR; KVÅ: AAA00, AAB00, AAB10, AAB20, AAB30, AAB99, AAC00, AAC05, AAC10, AAC15, AAC20, AAC30, AAC40, AAC99, AAD00, AAD05, AAD10, AAD15, AAD30, AAD40, AAD99, AAE00, AAE10, AAE20, AAE25, AAE30, AAE40, AAE50, AAE99, AAH10, AAH20, AAH30, AAH50, AAH60, AAH70, AAH80, AAH99, AAJ00, AAJ10, AAJ15, AAJ20, AAJ25, AAJ30, AAJ35, AAJ99, AAK00, AAK10, AAK50, AAK60, AAK80, AAK85, AAK99, AAL00, AAL10, AAL15, AAL20, AAL30, AAL40, AAL50, AAL99, AAM10, AAM30, AAM99, ABA00, ABB00, ABB02, ABB04, ABB10, ABB30, ABB40, ABB99, ABC10, ABC13, ABC16, ABC20-21, ABC23, ABC26, ABC28, ABC30, ABC33, ABC36, ABC40, ABC50, ABC53, ABC56, ABC60, ABC63, ABC66, ABC99, ABD10, ADA10, ADA20-21, ADA30-31, AWC00, AWE00, AWW99, BCA15, BCA20, BCA30-31, BCA40-41, BCA99, BDA10, BWA00, BWC00, BWE00, BWW99, DQB40, DQB50, EDB20, EJB30, EKB00, ENB20, ENB30, FAA00, FAA10, FAA96, FAB00, FAB10, FAB20, FAB30, FAB96, FAC00, FAC10, FAC20, FAC96, FAD00, FAD10, FAD96, FAE00, FAE10, FAE20, FAE30, FAE40, FAE50, FAE96, FAF00, FAF10, FAF20, FAF96, FAW96, FBA00, FBA10, FBA96, FBB00, FBB10, FBB20, FBB50, FBB96, FBC00, FBC10, FBC96, FBD00, FBD10, FBD20, FBD96, FBE00, FBE10, FBE20, FBE32, FBE35, FBE42, FBE96, FBF00, FBF10, FBF20, FBF96, FBG00, FBG10, FBG96, FBH00, FBH10, FBH96, FBJ00, FBJ10, FBJ96, FBK03, FBK13, FBK96, FBL00, FBL10, FBL20, FBL30, FBL40, FBL50, FBL96, FBM00, FBM10, FBM20, FBM30, FBM96, FBN00, FBN10, FBN96, FBW96, FCA20, FCA60, FCA70, FCA80, FCE00, FCE10, FCE20, FCE30, FCE40, FCE96, FDA00, FDA10, FDA96, FDB03, FDB20, FDB96, FDC00, FDC10, FDC20, FDC96, FDD00, FDD10, FDD13, FDD20, FDD96, FDF00, FDG00, FDG10, FDG96, FDH00, FDH10, FDH30, FDH40, FDH96, FDJ00, FDJ10, FDJ20, FDJ30, FDJ42, FDJ96, FDM00, FDM10, FDM11, FDM20, FDM96, FDW96, FEB00, FEB10, FEB51, FEB96, FEC00-01, FEC10, FEC96, FED00, FED03, FED10, FED96, FEE10, FEF00, FEF10, FEF20, FEF31, FEF96, FEW96, FFA10, FFA20, FFA30, FFB00, FFB10, FFB96, FFC00, FFC10, FFC50, FFC60, FFC96, FFD00, FFD20, FFD96, FFE00, FFE02, FFE10, FFE96, FFF00, FFF10, FFF20, FFF96, FFG00, FFG10, FFG20,	< 1 year

© 2024 Björklund J et al. *JAMA Network Open.*

Confounders	Source and definition	Time before index date
	FFG30, FFG96, FFH00, FFH10, FFH96, FFJ00, FFJ10, FFJ96, FFK10, FFK20, FFK96, FFL00, FFL10, FFL96, FFW96, FGA00, FGA10, FGA96, FGB00, FGB10, FGB96, FGC00, FGC10, FGC96, FGD00, FGD03, FGD10, FGD30, FGD40, FGD96, FGE00, FGE10, FGE20, FGE96, FGW96, FHA00, FHA10, FHA20, FHA96, FHB00, FHB10, FHB20, FHB30, FHB40, FHB50, FHB60, FHB70, FHB80, FHB96, FHC00, FHC10, FHC20, FHC30, FHC96, FHD00, FHD03, FHD10, FHD30, FHD96, FHE00, FHE10, FHE20, FHE30, FHE40, FHE96, FHF00, FHF10, FHF20, FHF30, FHF96, FHG00, FHG10, FHG20, FHG96, FHH00, FHH10, FHH20, FHH96, FHJ00, FHJ10, FHJ96, FHW96, FJB00, FJB10, FJB96, FJC00, FJC10, FJD00, FJD10, FJD20, FJD96, FJE00, FJE10, FJE20, FJE30, FJE96, FJF00, FJF10, FJF20, FJF96, FJW96, FKA00, FKA10, FKA20, FKA96, FKB00, FKB10, FKB96, FKC00, FKC10, FKC20, FKC30, FKC40, FKC50, FKC60, FKC96, FKD00, FKD10, FKD20, FKD96, FKW96, FLB00, FLB10, FLC00, FLC10, FLC96, FLD00, FLD10, FLD20, FLD30, FLD50, FLD96, FLE00, FLE10, FLE20, FLE96, FLF00, FLW96, FMA00, FMA10, FMA20, FMA96, FMB00, FMB10, FMB20, FMB96, FMC00, FMC10, FMC20, FMC96, FMD00, FMD10, FMD20, FMD30, FMD33, FMD40, FMD96, FMW96, FNA00, FNA10, FNA20, FNA96, FNB00, FNB20, FNB96, FNC10, FNC20, FNC30, FNC40, FNC50, FNC60, FNC96, FND10, FND20, FND96, FNE00, FNE10, FNE20, FNE96, FNF00, FNF10, FNF20, FNF30, FNF96, FNG00, FNG10, FNG20, FNG30, FNG96, FNH00, FNH10, FNH20, FNH96, FNJ00, FNJ10, FNJ96, FNK00, FNK10, FNK20, FNK96, FNW96, FPA00, FPA10, FPA20, FPA30, FPA40, FPA96, FPB96, FPC00, FPC10, FPC96, FPD00, FPD96, FPG10, FPH10, FPH20, FPW96, FQA00, FQA10, FQA20, FQA30, FQA40, FQA96, FQB00, FQB10, FQB20, FQB30, FQB96, FQW96, FWA00, FWC00, FWE00, FWF00, FWG00, FWG02, FWW96, FWW98, FXA00, FXA10, FXA13, FXA20, FXA96, FXB00, FXC00, FXD00, FXE00, FXF00, FXG00, FXH00, FXJ00, FXK00, FXN00, GAA97, GAB00, GAB10, GAB13, GAB20, GAB30, GAB96, GAC10-11, GAC20-21, GAC33-34, GAC37, GAC40-41, GAC43-44, GAC46-47, GAC53-54, GAC56-57, GAC96-98, GAD00, GAD03, GAD10, GAD96, GAE16, GAE20, GAE23, GAE26, GAE30, GAE40, GAE50, GAF00, GAF03, GAF10, GAF96, GAG10-11, GAG20-21, GAG30-31, GAG33, GAG36, GAG40, GAG50, GAG96-97, GAW96-97, GBA46, GBA50, GBA53, GBA96, GBC00, GBC03, GBC06, GBC10, GBC13, GBC96, GCA20, GCA26, GCA30, GCA40, GCA43, GCA50, GCA60, GCA70, GCA96, GDA00, GDA20-21, GDA30-31, GDA40-41, GDA96-97, GDB00-01, GDB10-11, GDB20-21, GDB96-97, GDC00-01, GDC10-11, GDC13, GDC20, GDC23, GDC26, GDC96-97, GDD00-01, GDD10-11, GDD20, GDD23, GDD26, GDD96-97, GDG00, GDG03, GDG10, GDG13, GDG30, GDG96, GDW96-97, GEA00, GEA10, GEA20, GEA96, GEB10, GEB20, GEB30, GEB40, GEB96, GEC10, GEC13-14, GEC20, GEC23-24, GEC96-97, GEW96, GWA00, GWC00, GWE00, GWF00, GWW96, HAB00, HAB40, HAB99, HAC10, HAC15, HAC20, HAC22, HAC25, HAC99, HAE00, HAE05, HAE10, HAE20, HAE99, HAF00, HWC00, HWE00, HWF00, HWW99, JAA13, JAA20-21, JAA23-24, JAA96-97, JAB00-01, JAB04, JAB10-11, JAB14, JAB20, JAB30, JAB40-41, JAB44, JAB50-51, JAB60-61, JAB70-71, JAB80-81, JAB84, JAB96-97, JAC10-11, JAC14, JAC20, JAC30, JAC40-41, JAC44, JAC50-51, JAC60-61, JAC70-71, JAC80-81, JAC84, JAC96-97, JAD10-11, JAD13, JAD20, JAD23, JAD30, JAD33, JAD40-41, JAD43, JAD47, JAD50-51, JAD60-61, JAD63, JAD67, JAD70-71, JAD73, JAD80-81, JAD84, JAD87, JAD96-97, JAE10-11, JAE20, JAE30, JAE40-41, JAE50-51, JAE60-61, JAE70-71, JAE80-81, JAE84, JAF11, JAF20, JAF30, JAF40-41, JAF50-51, JAF60-61, JAF70-71, JAF80-81, JAF84, JAF96-97, JAG00-01, JAG10-11, JAG20, JAG30, JAG40-41, JAG50-51, JAG60-61, JAG70-71, JAG80-81, JAG84, JAH00-01, JAH20-21, JAH30, JAH33, JAH40, JAK00-01, JAK03-04, JAK10, JAL00-01, JAL10-11, JAL20-21, JAL23, JAL30-31, JAL50-51, JAL96-97, JAM00, JAM10, JAP00-01, JAQ00, JAQ10, JAR10-11, JAR20, JAR30, JAR40-41, JAR50-51, JAR60-61, JAR70-71, JAR80-81, JAS10-11, JAS80-81, JAW96-97, JBA00-01, JBA20-21, JBB00-01, JBB10-11, JBB96-97, JBC00-01, JBW96-97, JCA00-01, JCA20, JCA60, JCA96-97, JCB00-01, JCC00, JCC10-11, JCC20, JCC30, JCC96-97, JCD00, JCD03, JCD10-11, JCD13, JCD20, JCD96-97, JCE00-01, JCE10, JCE20-21, JCE30, JCE33, JCE40, JCE50, JCE96-97, JCW96-97, JDA60-61, JDA63, JDB00-01, JDC00, JDC10-11, JDC20, JDC30, JDC40, JDC96-97, JDD00-01, JDD96, JDE00-01, JDE10, JDE20, JDE30-31, JDE96, JDF00-01, JDF10-11, JDF20-21, JDF40-41, JDF50-51, JDF60-61, JDF96-98, JDG00-01, JDG10-11, JDG96-97, JDH00, JDH40-41, JDH50,	

© 2024 Björklund J et al. *JAMA Network Open.*

Confounders	Source and definition	Time before index date
	JDH60-61, JDH63, JDH70-71, JDW10-11, JDW96-97, JEA00-01, JEA10, JEW96-97, JFA00, JFA10, JFA17, JFA60, JFA63, JFA70-71, JFA73-74, JFA76, JFA80-81, JFA83-84, JFA86, JFA96-97, JFB00-01, JFB10, JFB13, JFB20-21, JFB30-31, JFB33-34, JFB40-41, JFB43-44, JFB46-47, JFB50-51, JFB53-54, JFB60-61, JFB63-64, JFB96-97, JFC00-01, JFC10-11, JFC20-21, JFC30-31, JFC40-41, JFC50-51, JFC60-61, JFD00, JFD03-04, JFD10, JFD13, JFD20, JFD23, JFD96, JFE00, JFE96, JFF00-01, JFF10-11, JFF13, JFF16, JFF20-21, JFF23-24, JFF26-27, JFF30-31, JFF40-41, JFF50-51, JFF60, JFF96-97, JFG00, JFG10, JFG20, JFG23, JFG26, JFG29-30, JFG33, JFG36, JFG40, JFG50, JFG53, JFG56, JFG60, JFG70, JFG73, JFG76, JFG80, JFG83, JFG86, JFH00, JFH01, JFH10-11, JFH20, JFH30-31, JFH33, JFH40, JFH96, JFJ00-01, JFJ96-97, JFK00-01, JFK10, JFK20, JFK96-97, JFL00, JFL10-11, JFM00, JFW96-97, JGB00-01, JGB03-04, JGB10-11, JGB20, JGB30-31, JGB33-34, JGB36, JGB40, JGB50, JGB60-61, JGB96-97, JGC00-01, JGC40, JGC96-97, JGW96-97, JJA00, JJA10, JJA20-21, JJA23-24, JJA30-31, JJA40-41, JJA43-44, JJA50, JJA96-97, JJB00-01, JJB10-11, JJB20-21, JJB30-31, JJB40-41, JJB50-51, JJB53-54, JJB60-61, JJB71, JJB96-97, JJC00, JJC10, JJC20, JJC30, JJC40, JJC50, JJC60, JJC96, JJW96-97, JKA00, JKA10- 11, JKA20-21, JKA96-97, JKB00-01, JKB11, JKB40, JKB96-97, JKC00-01, JKC10, JKC20, JKC30, JKC40, JKC50, JKC96-97, JKD00-01, JKD10, JKD20, JKD30, JKD40, JKD50, JKD96, JKF00, JKF96-97, JKW96-97, JLA00, JLA10, JLA21, JLB00, JLB10, JLC00, JLC10-11, JLC20, JLC30, JLC40, JLC50, JLC96, JLD00, JLD10, JLD20, JLE00, JLE03, JLE10, JLE16, JLE20, JLE30, JLE40, JLE50, JLE56, JLE96, JLW96-97, JMA00, JMA10-11, JMA20, JMB00-01, JMB10, JMW96-97, JWA00, JWC00, JWE00, JWF00-01, JWW96-97, JXA00, JXA06, JXA10, JXA13, JXA16, KAA00, KAC00-01, KAC20-21, KAD00-01, KAD10, KAD40, KAD51, KAD96-97, KAE10, KAH00, KAH30, KAH40-41, KAH50, KAH70, KAH80-81, KAH96-97, KAJ10, KAJ96, KAS00, KAS10, KAS20, KAS40, KAS50, KAS60, KAS96, KAW96, KBA00-01, KBC00, KBD00, KBE96, KBF00-01, KBH00, KBH10, KBH20, KBH30, KBH40, KBH50, KBH96-97, KBJ00, KBJ10, KBJ20, KBJ40, KBJ60, KBJ70, KBJ96, KBV00, KBV10, KBW96-97, KCA00, KCC00, KCC10, KCC20, KCC30, KCC96, KCD10, KCD20, KCD40, KCF00, KCH00, KCH10, KCH20, KCH30, KCH33, KCH50, KCH60, KCH96, KCJ10, KCJ20, KCJ96, KDG10, KDG20, KDG50, KDH30, KDH50, KDH70, KDH96, KEA00, KEC00-01, KEC10, KEC20, KED00, KEV50, KEW96, KFC00, KFC10, KFH00, KGC00, KGC10, KKA00-01, KKA31, KKB10, KKF00, KKW96, KWA00, KWC00-01, KWE00-02, KWF00-01, KWW96, KXK00, KXK03, KXK06, LAA01, LAA31, LAA97, LAB00-01, LAC00-01, LAC10-11, LAC20-21, LAC30-31, LAC96-97, LAD00-01, LAE10-11, LAE20-21, LAF00-01, LAF10-11, LAF20, LAF30, LAG00-01, LAG10-11, LAG20-21, LAG96-97, LAW96-97, LBC10-11, LBC20-21, LBC96-97, LBD00-01, LBE00-01, LBF30-31, LBF40-41, LBF50-51, LBF60-61, LBF70, LBF96-97, LBW96-97, LCB00-01, LCB10-11, LCB14, LCB96-97, LCC00-01, LCC10-11, LCC96-97, LCD00-01, LCD04, LCD10-11, LCD30-31, LCD40, LCD96-97, LCE00, LCE10, LCE20, LCE96, LCF00-01, LCG10-11, LCG20-21, LCG30-31, LCG40-41, LCG96-97, LEE10, LEE20, LEE30, LEE40, LEE96, LEF13, LEF20, LEF23, LEF41, LEF50-51, LEF96-97, LEG20, LFC00, LFC96, LFD00, LFD10, LFE20, LFE96, LWA00, LWC00-01, LWE00-01, LWF00-01, MCA00, MCA10, MCA20, MCA30, MCA33, MCA96, MCB00, MCC00, MCW96, MWA00, MWC00, MWE00-01, MWF00-01, MWW96, NAB90-96, NAC90-96, NAG39, NAG49, NAG59, NAG69, NAG79, NAG89, NAG99, NAJ19, NAJ39, NAJ49, NAJ69, NAJ79, NAJ89, NAJ99, NAN09, NAN19, NAN29, NAN99, NAR09, NAR29, NAR39, NAR69, NAR79, NAT19, NAT29, NAW69, NAW89, NBB09, NBB19, NBB29, NBB39, NBB49, NBB59, NBB69, NBB99, NBC09, NBC19, NBC29, NBC39, NBC49, NBC59, NBC69, NBC99, NBG09, NBG19, NBG29, NBH22, NBH72, NBH92, NBJ19, NBJ39, NBJ49, NBJ59, NBJ69, NBJ79, NBJ89, NBJ99, NBN09, NBN19, NBN29, NBN39, NBN49, NBN99, NBP29, NBQ01-03, NBQ29, NBQ99, NBR09, NBR19, NBR29, NBR39, NBR49, NBR59, NBR69, NBR79, NBR89, NBR99, NBU09, NBU19, NBU29, NBW69, NBW89, NCB09, NCB19, NCB29, NCB39, NCB49, NCB59, NCB99, NCC09, NCC19, NCC29, NCC39, NCC49, NCC59, NCC99, NCJ39, NCJ49, NCJ59, NCJ69, NCJ79, NCJ89, NCJ99, NEH09, NEH99, NEJ19, NEJ39, NEJ49, NEJ69, NEJ79, NEJ89, NEJ99, NEN09, NEN19, NEN29, NEN99, NEP29, NEQ19, NEQ29, NEQ99, NER09, NER19, NER29, NER39, NER49, NER59, NER69, NER79, NER99, NEW69, NEW89, NFB09, NFB19, NFB29, NFB39, NFB49, NFB62, NFB99, NFC09, NFC19, NFC20-23, NFC29, NFC30-33, NFC39-43,	

© 2024 Björklund J et al. *JAMA Network Open.*

Confounders	Source and definition	Time before index date
	NFC49, NFC59, NFC99, NFG39, NFG49, NFG59, NFG99, NFH22, NFH72, NFH92, NFJ19, NFJ39, NFJ49, NFJ59, NFJ69, NFJ79, NFJ89, NFJ99, NFN09, NFN19, NFN29, NFN39, NFN49, NFN99, NFP29, NFQ09, NFQ19, NFQ29, NFQ99, NFR09, NFR19, NFR29, NFR39, NFR49, NFR59, NFR69, NFR79, NFR89, NFR99, NFS99, NFU09, NFU19, NFU39, NFU49, NFU89, NFW69, NFW89, NGB09, NGB19, NGB29, NGB39, NGB49, NGB53, NGB59, NGB99, NGC09, NGC19, NGC29, NGC39, NGC49, NGC53, NGC59, NGC99, NGH02, NGH22, NGH72, NGH92, NGJ19, NGJ39, NGJ49, NGJ59, NGJ69, NGJ79, NGJ89, NGJ99, NGN09, NGN19, NGN29, NGN39, NGN49, NGN99, NGP29, NGQ09, NGQ19, NGQ29, NGQ99, NGR09, NGR19, NGR29, NGR39, NGR49, NGR59, NGR69, NGR79, NGR99, NGU03, NGU09, NGU19, NGU49, NGU69, NGU89, NGW69, NGW89, NXW69, NXW89, PBB99, PHC23, PHC99, PJA10, PJD41-45, PJD52-55, PJD63-64, VAA00, VAA11, VAA22, VAA33, VAB00, VAB11, VAB22, VAB33, VAC48, VAG00, VAG11, VAG22, VAG33, VAH26, VAH37, VAJ00, VAJ11-14, VAJ16, VAJ19, VAJ22-24, VAJ29, VAJ33-35, VAJ39, VAK10-11, VAK20, VAK22, VAK30, VAK33, VAL00, VAL11, VAL22, VAL33, VAN00, VAN33, VAP00, VAP11, VAP22, VAP33, VAP48, VAQ00, VAQ11-14, VAQ22-24, VAQ26, VAQ33-35, VAQ37, VAT33, VAT48, VAU11, VAU22, VAU33, VAW00, VAW11, VAW22, VAW33, VBA00, VBA11, VBA22, VBA33, VBA44, VBB00, VBB11, VBB22, VBB33, VBB44, VBC00, VBC09, VBC33, VBE00, VBE11, VBE22, VBE33, VBE44, VBF11, VBF22, VBF33, VBF44, VBG00, VBG33, VBG44, VBH16, VBH26, VBH37, VBH56, VBJ00, VBJ12-15, VBJ23-25, VBJ34-35, VBK10, VBK20, VBK30, VBK40, VBL00, VBL11, VBL22, VBL33, VBL44, VBN00, VBN33, VBP00, VBP11, VBP22, VBP33, VBP44, VBQ00, VBQ12-16, VBQ23-26, VBQ34-36, VBR00, VBR11, VBR22, VBR33, VBR44, VBT00, VBT38, VBU00, VBU11, VBU22, VBU33, VBU44, VBW00, VBW11, VBW22, VBW33, VBW44, VCA00, VCA10-11, VCA22, VCA33, VCA44, VCA99, VCB00, VCB10-11, VCB22, VCB33, VCB44, VCB99, VCC00, VCC08, VCC10-11, VCC22-33, VCC44, VCC55, VCC99, VCE00, VCE10-11, VCE22, VCE33, VCE44, VCE99, VCF11, VCF22, VCF33, VCF44, VCF99, VCG00, VCG10-11, VCG22-23, VCG33, VCG44, VCG99, VCH11, VCH22, VCH33, VCH34, VCH44, VCH92, VCH99, VCJ00, VCJ10-11, VCJ17, VCJ19, VCJ22-23, VCJ26-27, VCJ29, VCJ33, VCJ39, VCJ44, VCJ59, VCJ99, VCK10, VCK20, VCK30, VCK33, VCK90, VCK99, VCL00, VCL10-11, VCL22, VCL33, VCL44, VCL99, VCN00, VCN10-11, VCN22-23, VCN33, VCN44, VCN99, VCP00, VCP10-11, VCP22-23, VCP33, VCP44, VCP99, VCQ00, VCQ10-11, VCQ22-23, VCQ33, VCQ44, VCQ99, VCR00, VCR10-11, VCR22, VCR33, VCR44, VCR99, VCS10-11, VCS22, VCS33, VCS44, VCS99, VCT08-09, VCU00, VCU10-11, VCU22-23, VCU33, VCU44, VCU99, VCW00, VCW10-11, VCW22-23, VCW33, VCW44, VCW99, VEA00, VEA11, VEA18, VEA22, VEA33, VEA38, VEA99, VEB00, VEB11, VEB18, VEB22, VEB33, VEB38, VEB44, VEB98, VEB99, VEC00, VEC11, VEC18, VEC22, VEC33, VEC38, VEC98-99, VED18, VED38, VED98, VEE00, VEE11, VEE18, VEE22, VEE33, VEE38, VEE44, VEE98-99, VEF11, VEF22, VEF33, VEF99, VEG00, VEG11, VEG18, VEG22, VEG33, VEG38, VEG44, VEG98-99, VEH11, VEH16, VEH22, VEH98, VEJ00, VEJ11, VEJ13-19, VEJ33-39, VEJ98-99, VEK10, VEK18, VEK20, VEK30, VEK38, VEK90, VEK98, VEL00, VEL11, VEL18, VEL22, VEL33, VEL38, VEL44, VEL98-99, VEN00, VEN11, VEN18, VEN22, VEN33, VEN38, VEN98, VEN99, VEP00, VEP11, VEP18, VEP22, VEP33, VEP38, VEP98-99, VEQ00, VEQ11, VEQ22, VEQ33, VEQ99, VER00, VER11, VER18, VER22, VER33, VER38, VER44, VER48, VER55, VER58, VER65, VER68, VER98-99, VET00, VET11, VET18, VET22, VET33, VET38, VET98, VET99, VEU00, VEU11, VEU18, VEU22, VEU33, VEU38, VEU44, VEU98, VEU99, VEW00, VEW11, VEW18, VEW22, VEW33, VEW38, VEW98-99, VGA00, VGA11, VGA23, VGA34, VGA45, VGA99, VGB00, VGB11, VGB23, VGB34, VGB45, VGB99, VGC00, VGC11, VGC23, VGC34, VGC45, VGC67, VGC99, VGE00, VGE11, VGE23, VGE34, VGE45, VGE99, VGF11, VGF23, VGF34, VGF45, VGF99, VGG00, VGG11, VGG23, VGG34, VGG45, VGG99, VGH39, VGH49, VGH99, VGJ00, VGJ11, VGJ19, VGJ23, VGJ29, VGJ34, VGJ39, VGJ45, VGJ49, VGJ99, VGK10, VGK20, VGK30, VGK40, VGK60, VGK90, VGL00, VGL11, VGL23, VGL34, VGL45, VGL99, VGN00, VGN11, VGN23, VGN34, VGN45, VGN67, VGN99, VGP00, VGP11, VGP23, VGP34, VGP45, VGP67, VGP99, VGQ00, VGQ11, VGQ23, VGQ34, VGQ45, VGQ67, VGQ99, VGR00, VGR11, VGR23, VGR34, VGR45, VGR67, VGR99, VGT00, VGT11, VGT23, VGT34, VGT45, VGT67, VGT99, VGU00, VGU11, VGU23, VGU34, VGU45, VGU67, VGU99, VGW00, VGW11, VGW23, VGW34,	

© 2024 Björklund J et al. *JAMA Network Open.*

Confounders	Source and definition	Time before index date
	VGW45, VGW99, VHA00, VHA11, VHA21, VHA31, VHA99, VHB00, VHB11, VHB21, VHB31, VHB99, VHC00, VHC11, VHC21, VHC31, VHC99, VHE00, VHE11, VHE21, VHE31, VHE99, VHF11, VHF21, VHF31, VHF99, VHG00, VHG11, VHG21, VHG31, VHG99, VHJ00, VHJ11-16, VHJ21-24, VHJ31, VHJ99, VHK10, VHK20, VHK30, VHK90, VHL00, VHL11, VHL21, VHL31, VHL99, VHN00, VHN11, VHN21, VHN31, VHN99, VHP00, VHP11, VHP21, VHP31, VHP99, VHQ00, VHQ11, VHQ21, VHQ31, VHQ99, VHR00, VHR11, VHR21, VHR31, VHR99, VHS00, VHS11, VHS21, VHS31, VHS99, VHT00, VHT11, VHT21, VHT31, VHT99, VHU00, VHU11, VHU21, VHU31, VHU99, VHW00, VHW11, VHW21, VHW31, VHW99, VKA00, VKA11, VKA18, VKA22, VKA33, VKB00, VKB11, VKB18, VKB22, VKB33, VKC00, VKC11, VKC18, VKC22, VKC33, VKD18, VKE00, VKE11, VKE18, VKE22, VKE33, VKF11, VKF22, VKF33, VKG00, VKG11, VKG18, VKG22, VKG33, VKH18, VKH22-23, VKH33, VKJ00, VKJ11-13, VKJ15, VKJ18-19, VKJ22-24, VKJ29, VKJ33-34, VKJ39, VKJ44, VKK10, VKK18, VKK20, VKK22-23, VKK30, VKK33, VKK40, VKL00, VKL11, VKL18, VKL22, VKL33, VKN00, VKN11, VKN18, VKN22-23, VKN33, VKP00, VKP11, VKP18, VKP22-23, VKP33, VKQ00, VKQ11, VKQ18, VKQ22-23, VKQ33, VKR00, VKR11, VKR18, VKR22, VKR33, VKR44, VKS00, VKS11, VKS18, VKS22, VKS33, VKT00, VKT11, VKT18, VKT22, VKT33, VKU00, VKU11, VKU18, VKU22, VKU33, VKW00, VKW11, VKW18, VKW22, VKW33, VME30, VME40, VMG31, VMG41, VMJ31, VMJ41, VML30, VML40, VMN30, VMN40, VMP31, VMP41, VMQ31, VMQ41, VMR30, VMR40, VMU30, VMU40, VMW30, VMW40, VPA00, VPA10, VPA20, VPA90, VPB00, VPB10, VPB20, VPB90, VPC01, VPC11, VPC21, VPC91, VPE00, VPE10, VPE20, VPE90, VPG01, VPG11, VPG21, VPG91, VPH13, VPJ01, VPJ11, VPJ21, VPJ91, VPK11-12, VPK21, VPK91-92, VPL00, VPL10, VPL20, VPL90, VPN00, VPN10, VPN20, VPN90, VPP01, VPP11, VPP21, VPP91, VPQ01, VPQ11, VPQ12, VPQ21, VPQ91, VPR00, VPR10, VPR20, VPR90, VPU00, VPU10, VPU20, VPU90, VPW00, VPW10, VPW20, VPW90, VQA00, VQA10, VQA20, VQA90, VQB00, VQB10, VQB20, VQB50, VQB90, VQC01, VQC11, VQC21, VQC51, VQC91, VQE00, VQE10, VQE20, VQE90, VQF10, VQF20, VQF90, VQG01, VQG11, VQG21, VQG91, VQJ11, VQJ21, VQJ91, VQK11-12, VQK21-22, VQK52, VQK91-92, VQL00, VQL10, VQL20, VQL90, VQN00, VQN10, VQN20, VQN90, VQP01, VQP11-12, VQP21, VQP91, VQQ01, VQQ11, VQQ21, VQQ91, VQR00, VQR10, VQR20, VQR90, VQS00, VQS10, VQS20, VQS90, VQT00, VQT20, VQT50, VQT90, VQU00, VQU10, VQU20, VQU90, VQW00, VQW10, VQW20, VQW90, VSA00, VSA10, VSA20, VSE00, VSE10, VSE20, VSE90, VSF10, VSF20, VSG01, VSG11-12, VSG21, VSJ11-12, VSJ21, VSK12, VSP01, VSP11, VSP13, VSP21, VSQ01, VSQ11, VSQ21, VSR00, VSR10, VSR20, VSR90, VSU00, VSU10, VSU20, VSW00, VSW10, VSW20, VSW90, VUC11, VUC21, VUC31, VUC41, VUC51, VUC91, VUH12, VUK14, VUK24, VUK34, VUL10, VUL20, VUL30, VUL40, VUL50, VUL90, VWA00, VWC00, VWE00, VWF00, VWG00, VWH00, VWW00, VXK11, VXK16, VXK21, VXK26, VXK31, VXK36-37, VXK41, VXK46-47, VXK51, VXK56, VXK61, VXK66-67, YGA10, YGA20, YJA00, YKA00-01, ZZN00, ZZN10, ZZN20, ZZP00, ZZP10, ZZP20, ZZR05, ZZR10, ZZR20, ZZR30, ZZR40, ZZR45, ZZR60, ZZS00, ZZS10, ZZS20	
Demographic information		
Age at surgery	TPR; Categorized as ≤49, 50-59, 60-69, 70-79, ≥80	At index date
Sex	TPR;	At index date
Region of residence	TPR; Region of hospital in which index surgery was performed (out of Sweden's 6 health care regions)	At index date

Abbreviations: NPR, The National Patient Register; ICD-10, International Classification of Disease (Swedish version); KVÅ, Klassifikation av vårdåtgärder, an adapted version of NOMESCO classification of surgical procedures; TPR, Total Population Register

[1] All the ICD codes in the National patient register were available as three-digit codes, except for the outcome related ICD codes which were available as four-digit codes

[2] Comorbidities were identified as the main or any contributory diagnoses from 1 year before the index date until, but not including the index date. This definition likely led to underestimation of the incidence of chronic diseases commonly managed in primary care and do not require hospitalization or admission to outpatient care such as hypertension and diabetes mellitus. It is, however, likely that such comorbidities could be recorded as

© 2024 Björklund J et al. *JAMA Network Open.*

Confounders	Source and definition	Time before index date

one of the contributory diagnoses in NPR even when subjects were hospitalized for some unrelated reason. Since all cancer surgery patients were either hospitalized or admitted to the outpatient care on the index date, to allow for the non-differential misclassification of the confounders between subjects in the cancer surgery cohort and the comparison cohort, we did not include index date in the confounder definition.

© 2024 Björklund J et al. *JAMA Network Open.*

eTable 4. Patient Demographics for Separate Tumor Forms

Cancer type	Cohort	Sample size	Age	Age groups (%)					Sex		Hosp duration[1]	Intra-hospitalization		Post-hospitalization		All-cause mortality	Death with PE[2]
				≤49	50-59	60-69	70-79	≥80	Female	Male		PE	DVT	PE	DVT		
		N	median (IQR)	%	%	%	%	%	%	%	median (IQR)	%	%	%	%	%	%
Bladder	Cancer surgery	8 472	69 (63, 75)	3.80	12.42	35.30	40.33	8.14	24.42	75.58	17 (13, 23)	0.89	0.71	2.92	5.00	20.83	0.66
	Comparison	77 260	69 (62, 74)	4.13	13.24	36.33	38.96	7.34	24.88	75.12	N/A	0.01	0.02	0.23	0.33	2.31	0.06
Breast	Cancer surgery	162 883	63 (53, 73)	17.59	22.43	27.45	20.39	12.14	99.47	0.53	2 (1, 4)	0.03	0.03	0.75	1.62	2.86	0.11
	Comparison	1 533 875	63 (52, 72)	18.39	23.06	27.44	19.72	11.39	99.49	0.51	N/A	0.00	0.00	0.16	0.26	1.69	0.05
Colorectal	Cancer surgery	113 484	73 (65, 80)	4.46	10.02	23.44	35.60	26.49	48.46	51.54	11 (8, 16)	0.53	0.35	1.84	2.52	16.07	0.43
	Comparison	1 024 798	72 (64, 79)	4.88	10.77	24.35	35.11	24.88	49.19	50.81	N/A	0.01	0.02	0.27	0.37	3.87	0.09
Gynecologic	Cancer surgery	57 654	65 (55, 74)	15.93	19.54	28.12	25.37	11.04	N/A	N/A	7 (5, 9)	0.29	0.25	1.49	2.28	8.27	0.33
	Comparison	541 225	64 (54, 73)	16.75	20.11	28.14	24.57	10.42	N/A	N/A	N/A	0.00	0.01	0.18	0.26	1.64	0.05
Kidney and UTUC	Cancer surgery	20 893	68 (59, 75)	9.13	15.88	30.04	33.43	11.53	40.53	59.47	8 (6, 11)	0.49	1.14	1.58	2.46	13.48	0.33
	Comparison	192 773	68 (59, 74)	9.79	16.72	30.60	32.34	10.56	40.84	59.16	N/A	0.01	0.01	0.20	0.32	2.20	0.06
Lung	Cancer surgery	13 890	67 (60, 72)	7.16	16.75	37.93	34.08	4.07	47.33	52.67	7 (6, 10)	0.29	0.14	2.78	1.68	14.88	0.87
	Comparison	128 943	67 (59, 72)	7.61	17.50	38.37	32.82	3.71	47.80	52.2	N/A	0.00	0.01	0.16	0.27	1.60	0.04
Prostate	Cancer surgery	39 921	64 (60, 68)	1.53	21.05	60.68	16.62	0.11	N/A	N/A	3 (2, 6)	0.12	0.07	0.75	1.07	0.51	0.06
	Comparison	372 816	64 (60, 68)	1.62	21.90	60.65	15.74	0.09	N/A	N/A	N/A	0.00	0.00	0.17	0.32	1.18	0.02
Gastroeso-phageal	Cancer surgery	15 021	71 (62, 77)	5.96	13.48	26.77	36.83	16.96	35.18	64.82	16 (11, 24)	1.01	0.53	2.38	2.52	33.64	0.71
	Comparison	137 653	70 (61, 77)	6.44	14.34	27.50	35.98	15.75	35.50	64.5	N/A	0.02	0.01	0.25	0.32	3.11	0.10

Abbreviations: IQR, Interquartile range; PE, Pulmonary embolism; DVT, deep vein thrombosis; UTUC, Upper tract urothelial cancer; N/A, not applicable

[1] When the date of discharge from the hospital was the same as the date of admission to the hospital, hospitalization duration equals 0

[2] Defined as pulmonary embolism registered in the Cause of Death Register as an underlying or one of the first three contributing diagnoses

© 2024 Björklund J et al. *JAMA Network Open.*

eTable 5. The Hazard Ratios and the 95% Confidence Intervals of Deep Vein Thrombosis After the Discharge From the Hospital for Cancer Surgery vs Comparison Cohort, by Cancer Type

Cancer type		Cancer surgery vs comparison cohort			Sex[2]	Age				
		At 30 days	At 90 days	At 365 days	Female	≤49	50-59	60-69	70-79	≥80
Bladder	HR[1]	28.25	19.17	7.77	1.00	0.60	0.89	REF	1.14	1.26
	95% CI	19.37, 41.19	14.08, 26.09	5.42, 11.14	0.83, 1.21	0.35, 1.03	0.67, 1.17		0.95, 1.37	0.93, 1.70
Breast	HR[1]	10.04	9.24	2.31	1.02	0.60	0.65	REF	1.35	1.75
	95% CI	9.00, 11.21	8.63, 9.89	2.02, 2.64	0.75, 1.40	0.55, 0.66	0.60, 0.71		1.26, 1.44	1.63, 1.88
Colorectal	HR[1]	11.38	8.84	2.75	1.00	0.56	0.70	REF	1.10	1.24
	95% CI	10.16, 12.65	8.17, 9.55	2.39, 3.16	0.95, 1.05	0.48, 0.66	0.63, 0.78		1.03, 1.18	1.16, 1.34
Gynecological	HR[1]	13.78	10.25	4.29	N/A	0.51	0.72	REF	1.39	1.65
	95% CI	11.68, 16.25	9.01, 11.65	3.59, 5.13	N/A	0.43, 0.59	0.63, 0.81		1.26, 1.54	1.46, 1.86
Kidney and UTUC	HR[1]	9.53	4.16	2.39	0.89	0.30	0.88	REF	1.45	1.90
	95% CI	7.10, 12.80	3.29, 5.27	1.69, 3.39	0.78, 1.03	0.19, 0.46	0.70, 1.10		1.23, 1.71	1.55, 2.34
Lung	HR[1]	10.22	7.76	3.06	0.95	0.72	0.71	REF	1.09	1.21
	95% CI	7.13, 14.65	6.00, 10.04	2.04, 4.60	0.80, 1.12	0.49, 1.06	0.54, 0.93		0.90, 1.31	0.81, 1.80
Prostate	HR[1]	10.05	2.58	0.79	N/A	0.41	0.59	REF	1.12	0.55
	95% CI	8.20, 12.31	2.01, 3.31	0.56, 1.12	N/A	0.22, 0.74	0.50, 0.68		0.98, 1.27	0.08, 3.89
Gastroesophageal	HR[1]	12.28	7.63	4.28	0.98	0.52	0.68	REF	1.29	1.35
	95% CI	8.96, 16.81	5.91, 9.85	2.92, 6.27	0.84, 1.15	0.33, 0.80	0.51, 0.90		1.07, 1.55	1.08, 1.70

Abbreviations: UTUC, Upper tract urothelial cancer; HR, Hazard ratio; CI, Confidence interval; N/A, not applicable; REF, Reference

[1] All models adjusted for the matching variables, other major surgery, and comorbidities (ischemic heart disease, peripheral vascular disease, cardiac arrhythmia, cerebrovascular disease, congestive heart failure, paralysis, hypertension, diabetes mellitus, chronic pulmonary disease, renal disease, and anemia)

[2] Male sex as a reference

© 2024 Björklund J et al. *JAMA Network Open.*

eTable 6. Sensitivity Analysis Restricted to Subjects Operated From 2002 Onwards: Crude Absolute Risk of Pulmonary Embolism and Deep Vein Thrombosis 30, 90 and 365 Days From Index Date, by Cancer Type

			Cancer type							
			Bladder	Breast	Colorectal	Gynecological	Kidney/UTUC	Lung	Prostate	GE
30 days	PE	$AR_{operated}$	1.23	0.11	0.65	0.57	0.76	0.61	0.39	1.38
		$AR_{matched}$	0.02	0.01	0.02	0.02	0.01	0.01	0.02	0.02
		ARD	1.22	0.10	0.62	0.55	0.75	0.60	0.37	1.37
		95% CI (ARD)	0.91, 1.53	0.08, 0.12	0.56, 0.69	0.47, 0.64	0.59, 0.91	0.43, 0.77	0.31, 0.44	1.04, 1.69
	DVT	$AR_{operated}$	0.93	0.16	0.46	0.48	1.78	0.32	0.43	0.73
		$AR_{matched}$	0.03	0.02	0.03	0.03	0.02	0.03	0.03	0.04
		ARD	0.89	0.14	0.43	0.45	1.76	0.29	0.41	0.70
		95% CI (ARD)	0.62, 1.16	0.11, 0.16	0.37, 0.48	0.38, 0.53	1.52, 2.00	0.16, 0.41	0.34, 0.48	0.46, 0.93
		% excluded[1]	7.14	0.04	4.00	0.76	1.51	0.83	0.08	13.14
90 days	PE	$AR_{operated}$	2.13	0.27	1.12	1.08	1.14	1.25	0.53	2.08
		$AR_{matched}$	0.06	0.04	0.06	0.05	0.05	0.04	0.05	0.05
		ARD	2.07	0.23	1.06	1.03	1.09	1.21	0.48	2.03
		95% CI (ARD)	1.67, 2.46	0.19, 2.26	0.97- 1.14	0.91, 1.15	0.89, 1.28	0.97, 1.46	0.41, 0.56	1.66, 2.40
	DVT	$AR_{operated}$	2.55	0.83	1.18	1.17	2.19	0.87	0.69	1.35
		$AR_{matched}$	0.08	0.07	0.10	0.07	0.07	0.09	0.08	0.11
		ARD	2.47	0.76	1.08	1.10	2.12	0.78	0.61	1.24
		95% CI (ARD)	2.04, 2.89	0.70, 0.82	0.99, 1.16	0.98, 1.22	1.85, 2.38	0.58, 0.99	0.52, 0.70	0.94, 1.54
		% excluded[1]	0.32	0.00	0.17	0.02	0.04	0.01	0.00	0.82
365 days	PE	$AR_{operated}$	3.38	0.87	2.26	1.93	1.84	3.06	0.72	3.16
		$AR_{matched}$	0.25	0.17	0.26	0.20	0.20	0.19	0.18	0.26
		ARD	3.13	0.70	2.00	1.73	1.63	2.88	0.54	2.90
		95% CI (ARD)	2.64, 3.62	0.64, 0.76	1.88, 2.12	1.58, 1.89	1.39, 1.88	2.50, 3.26	0.45, 0.63	2.45, 3.35
	DVT	$AR_{operated}$	5.33	1.93	2.81	2.56	3.02	1.86	1.03	2.56
		$AR_{matched}$	0.34	0.28	0.39	0.29	0.34	0.32	0.33	0.40
		ARD	4.98	1.65	2.42	2.27	2.68	1.54	0.70	2.16
		95% CI (ARD)	4.37, 5.59	1.56, 1.74	2.29, 2.56	2.09, 2.45	2.37, 2.99	1.24, 1.84	0.59, 0.81	1.75, 2.57
		% excluded[1]	0.00	0.00	0.00	0.00	0.01	0.00	0.00	0.00

Abbreviations: UTUC, Upper tract urothelial cancer; GE, Gastroesophageal; PE, Pulmonary embolism; DVT, deep vein thrombosis; AR, Absolute risk in percent; ARD, absolute risk difference in percentage units; CI, Confidence interval

[1] Percentage of patients who were still hospitalized for the index hospitalization at the time of analysis

© 2024 Björklund J et al. *JAMA Network Open.*

eTable 7. Sensitivity Analysis Restricted to Subjects Operated From 2002 Onwards: Adjusted Odds Ratios and 95% Confidence Intervals of Pulmonary Embolism and Deep Vein Thrombosis During Hospitalization for Cancer Surgery vs Comparison Cohort, by Cancer Type

Cancer type	PE		DVT	
	OR[1]	95 % CI	OR[1]	95 % CI
Bladder	104.46	37.38, 291.94	35.81	15.25, 84.1 1
Breast	34.45	15.74, 75.41	9.20	5.14, 16.48
Colorectal	53.10	39.46, 71.46	15.35	11.75, 20.06
Gynecologic	88.98	47.78, 165.72	47.37	24.59, 91.23
Kidney and UTUC	91.45	39.30, 212.83	220.31	108.13, 448.85
Lung	50.55	14.22, 179.67	12.97	4.35, 38.70
Prostate	68.70	29.65, 159.19	12.57	6.31, 25.06
Gastroesophageal	128.06	55.18, 297.22	32.70	15.58, 68.60

Abbreviations: PE, pulmonary embolism; DVT, deep vein thrombosis; OR, odds ratios; CIs, Confidence interval; UTUC, upper tract urothelial cancer

[1] All models adjusted for the matching variables, other major surgery, and comorbidities (ischemic heart disease, peripheral vascular disease, cardiac arrhythmia, cerebrovascular disease, congestive heart failure, paralysis, hypertension, diabetes mellitus, chronic pulmonary disease, renal disease, and anemia)

© 2024 Björklund J et al. *JAMA Network Open.*

eTable 8. Sensitivity Analysis Restricted to Subjects Operated From 2002 Onwards: Hazard Ratios and the 95% Confidence Intervals of Pulmonary Embolism and Deep Vein Thrombosis After the Discharge From the Hospital for Cancer Surgery vs Comparison Cohort, by Cancer Type

Cancer type		PE			DVT		
		30 days	90 days	365 days	30 days	90 days	365 days
Bladder	HR[1]	21.03	7.09	7.55	32.41	20.51	8.56
	95% CI	12.56, 38.65	4.15, 12.56	4.36, 13.08	20.32, 51.69	14.31, 29.41	5.51, 13.30
Breast	HR[1]	5.58	6.37	2.26	12.30	11.34	1.99
	95% CI	4.60, 6.77	5.66, 7.17	1.83, 2.79	10.72, 14.11	10.43, 12.34	1.67, 2.37
Colorectal	HR[1]	12.09	9.69	3.15	10.99	11.16	2.65
	95% CI	10.09, 14.48	8.60, 10.91	2.53, 3.92	9.48, 12.74	10.13, 12.28	2.21, 3.19
Gynecological	HR[1]	17.23	11.75	3.20	15.72	10.82	4.35
	95% CI	13.29, 22.33	9.59, 14.40	2.38, 4.32	12.69, 19.48	9.21, 12.71	3.43, 5.50
Kidney and UTUC	HR[1]	14.42	4.80	4.36	10.67	4.83	2.30
	95% CI	9.25, 22.46	3.21, 7.19	2.74, 6.92	7.15, 15.94	3.61, 6.46	1.42, 3.72
Lung	HR[1]	33.28	21.72	8.70	9.63	8.26	2.23
	95% CI	20.27, 54.63	15.40, 30.63	5.37, 14.12	6.25, 14.86	6.02, 11.35	1.31, 3.81
Prostate	HR[1]	11.90	1.60	1.37	9.40	2.68	0.90
	95% CI	8.95, 15.82	1.00, 2.55	0.91, 2.06	7.57, 11.66	2.09, 3.44	0.63, 1.28
Gastroesophageal	HR[1]	14.96	7.62	6.83	10.07	5.72	3.85
	95% CI	8.30, 26.99	4.91, 11.83	3.77, 12.36	6.29, 16.12	3.93, 8.31	2.17, 6.82

Abbreviations: PE, Pulmonary embolism; DVT, Deep vein thrombosis; UTUC, Upper tract urothelial cancer; HR, Hazard ratio; CI, Confidence interval

[1] All models adjusted for the matching variables, other major surgery, and comorbidities (ischemic heart disease, peripheral vascular disease, cardiac arrhythmia, cerebrovascular disease, congestive heart failure, paralysis, hypertension, diabetes mellitus, chronic pulmonary disease, renal disease, and anemia)

© 2024 Björklund J et al. *JAMA Network Open.*

eFigure 2. The Plot of Hazard Ratios and Corresponding 95% Confidence Intervals of Pulmonary Embolism and Deep Vein Thrombosis After the Discharge From the Hospital for Cancer Surgery vs Comparison Cohort, by Cancer Type

Notes: Y-axis presented in logarithmic scale and truncated at 64 if hazard ratios or upper confidence intervals exceeded 64. All models adjusted for the matching variables, other major surgery, and comorbidities (ischemic heart disease, peripheral vascular disease, cardiac arrhythmia, cerebrovascular disease, congestive heart failure, paralysis, hypertension, diabetes mellitus, chronic pulmonary disease, renal disease, and anemia)
Abbreviations: UTUC, upper tract urothelial cancer

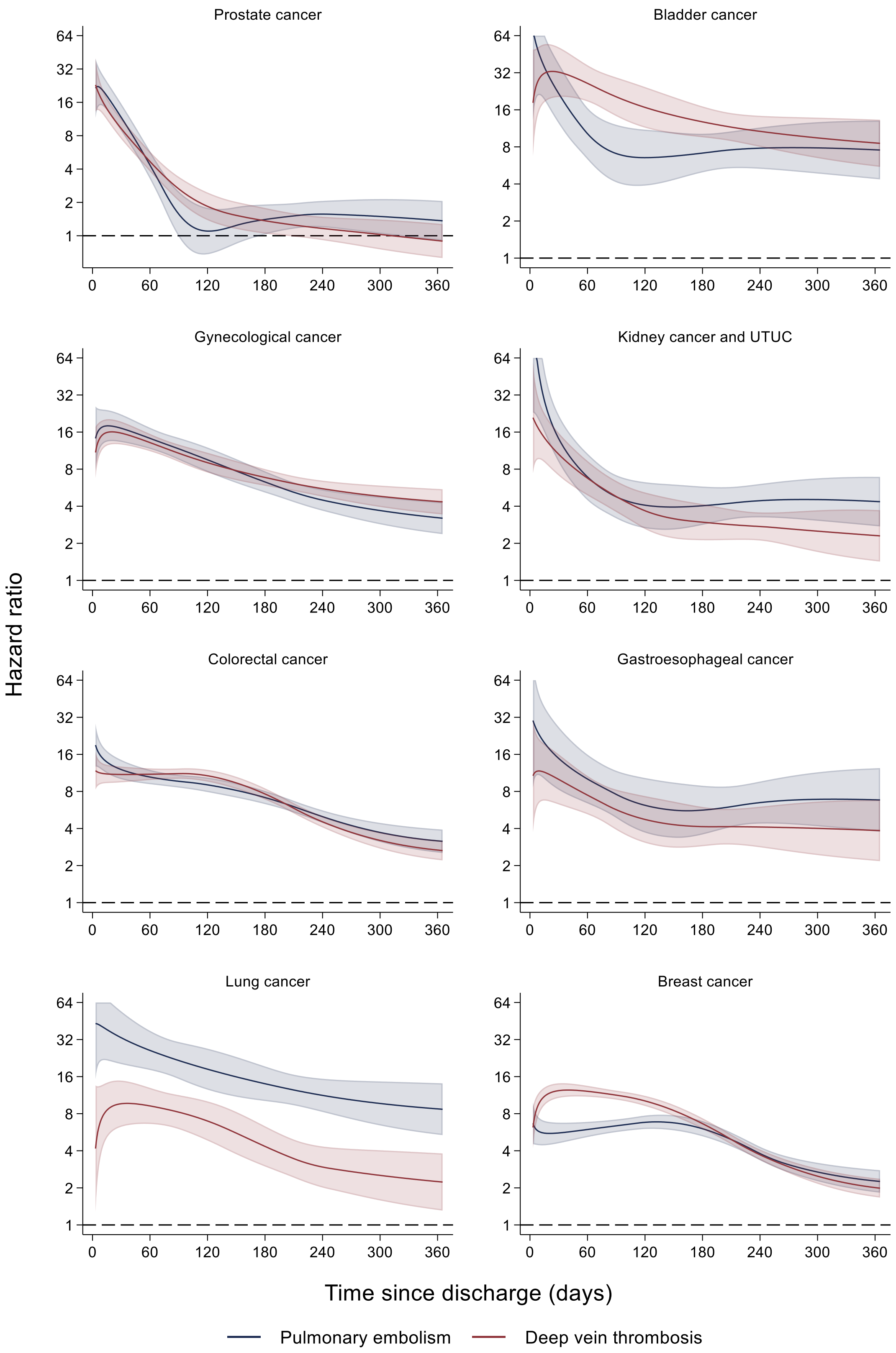

© 2024 Björklund J et al. *JAMA Network Open.*

eTable 9. Sensitivity Analysis in Which Pulmonary Embolism Was Defined as a Main Inpatient Diagnosis or Underlying Cause of Death: Crude Absolute Risk of Pulmonary Embolism 30, 90 and 365 Days From Index Date, by Cancer Type

		Cancer type							
		Bladder	Breast	Colorectal	Gynecological	Kidney/UTUC	Lung	Prostate	GE
30 days	$AR_{operated}$	0.43	0.06	0.18	0.15	0.22	0.30	0.23	0.11
	$AR_{matched}$	0.02	0.01	0.01	0.01	0.01	0.01	0.01	0.01
	ARD	0.41	0.05	0.17	0.15	0.21	0.29	0.22	0.10
	95% CI (ARD)	0.26, 0.56	0.04, 0.06	0.15, 0.20	0.11, 0.18	0.15, 0.28	0.20, 0.38	0.17, 0.27	0.04, 0.16
	% excluded[1]	12.04	0.10	5.57	1.32	2.47	0.95	0.12	15.09
90 days	$AR_{operated}$	0.78	0.15	0.37	0.33	0.35	0.60	0.34	0.33
	$AR_{matched}$	0.04	0.03	0.04	0.03	0.03	0.02	0.03	0.04
	ARD	0.74	0.12	0.33	0.30	0.33	0.58	0.32	0.29
	95% CI (ARD)	0.55, 0.93	0.10, 0.14	0.30, 0.37	0.25, 0.35	0.25, 0.41	0.45, 0.71	0.26, 0.37	0.20, 0.38
	% excluded[1]	0.43	0.01	0.22	0.03	0.05	0.01	0.00	0.72
365 days	$AR_{operated}$	1.26	0.51	0.83	0.66	0.64	1.48	0.48	0.69
	$AR_{matched}$	0.15	0.11	0.16	0.11	0.12	0.11	0.11	0.16
	ARD	1.12	0.41	0.67	0.55	0.52	1.38	0.37	0.53
	95% CI (ARD)	0.88, 1.35	0.37, 0.44	0.61, 0.72	0.49, 0.62	0.41, 0.62	1.18, 1.58	0.31, 0.44	0.40, 0.67
	% excluded[1]	0.00	0.00	0.00	0.00	0.00	0.00	0.00	0.00

Abbreviations: UTUC, Upper tract urothelial cancer; GE, Gastroesophageal; AR, Absolute risk in percent; ARD, absolute risk difference in percentage units; CI, Confidence intervals

[1] Percentage of patients who were still hospitalized for the index hospitalization at the time of analysis

© 2024 Björklund J et al. *JAMA Network Open.*

eTable 10. Sensitivity Analysis in Which Pulmonary Embolism Was Defined as a Main Inpatient Diagnosis or Underlying Cause of Death: Odds Ratios and the 95% Confidence Intervals of Pulmonary Embolism During Hospitalization for Cancer Surgery vs Comparison Cohort, by Cancer Type

Cancer type	OR[1]	95 % CI
Bladder	14.27	5.63, 36.21
Breast	7.50	2.76, 20.34
Colorectal	8.52	5.64, 12.88
Gynecologic	13.87	6.57, 29.28
Kidney and UTUC	12.80	4.41, 37.12
Lung	17.23	4.38, 67.83
Prostate	18.15	5.60, 58.85
Gastroesophageal	5.42	1.86, 15.81

Abbreviations: OR, odds ratios; CI, Confidence interval; UTUC, upper tract urothelial cancer

[1] All models adjusted for the matching variables, other major surgery, and comorbidities (ischemic heart disease, peripheral vascular disease, cardiac arrhythmia, cerebrovascular disease, congestive heart failure, paralysis, hypertension, diabetes mellitus, chronic pulmonary disease, renal disease, and anemia)

© 2024 Björklund J et al. *JAMA Network Open.*

eTable 11. Sensitivity Analysis in Which Pulmonary Embolism Was Defined as a Main Inpatient Diagnosis or Underlying Cause of Death: Hazard Ratios and the 95% Confidence Intervals of Pulmonary Embolism After the Discharge From the Hospital for Cancer Surgery vs Comparison Cohort, by Cancer Type

Cancer type		30 days	90 days	365 days
Bladder	HR[1]	19.97	5.64	4.49
	95% CIs	10.87, 36.69	2.90, 10.87	2.38, 8.48
Breast	HR[1]	5.28	5.94	2.58
	95% CIs	4.39, 6.35	5.30, 6.67	2.11, 3.15
Colorectal	HR[1]	10.00	6.08	1.94
	95% CIs	8.44, 11.85	5.33, 6.95	1.52, 2.49
Gynecologic	HR[1]	13.98	7.21	2.56
	95% CIs	10.78, 18.13	5.82, 8.94	1.88, 3.49
Kidney and UTUC	HR[1]	13.41	3.26	2.93
	95% CIs	8.44, 21.32	2.02, 5.24	1.82, 4.72
Lung	HR[1]	28.89	17.13	7.21
	95% CIs	17.30, 48.25	12.10, 24.25	4.30, 12.11
Prostate	HR[1]	16.99	1.86	1.82
	95% CIs	12.31, 23.46	1.14, 3.03	1.17, 2.83
Gastroesophageal	HR[1]	13.05	6.42	2.00
	95% CIs	8.08, 21.08	4.23, 9.74	1.02, 3.93

Abbreviations: UTUC, Upper tract urothelial cancer; HR, Hazard ratio; CI, Confidence interval
[1] All models adjusted for the matching variables, other major surgery, and comorbidities (ischemic heart disease, peripheral vascular disease, cardiac arrhythmia, cerebrovascular disease, congestive heart failure, paralysis, hypertension, diabetes mellitus, chronic pulmonary disease, renal disease, and anemia)

© 2024 Björklund J et al. *JAMA Network Open.*

eFigure 3. Sensitivity Analysis in Which Pulmonary Embolism Was Defined as a Main Inpatient Diagnosis or Underlying Cause of Death: the Plot of Hazard Ratios and Corresponding 95% Confidence Intervals of Pulmonary Embolism After the Discharge From the Hospital for Cancer Surgery vs Comparison Cohort, by Cancer Type

Notes: Y-axis presented in logarithmic scale and truncated at 64 if hazard ratios or upper confidence intervals exceeded 64. All models adjusted for the matching variables, other major surgery, and comorbidities (ischemic heart disease, peripheral vascular disease, cardiac arrhythmia, cerebrovascular disease, congestive heart failure, paralysis, hypertension, diabetes mellitus, chronic pulmonary disease, renal disease, and anemia).
Abbreviations: UTUC, Upper tract urothelial cancer

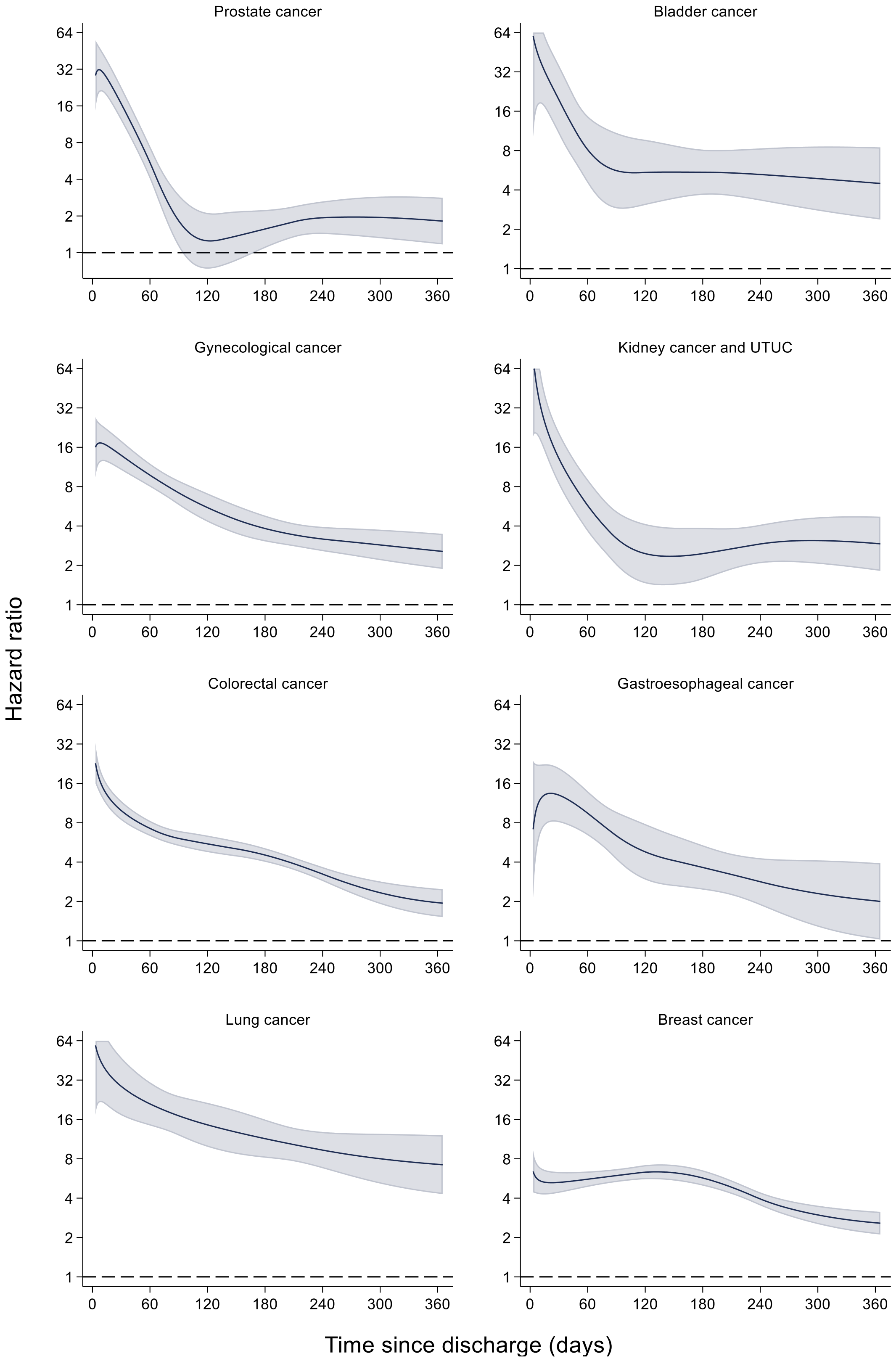

© 2024 Björklund J et al. *JAMA Network Open.*

eFigure 4. Sensitivity Analysis Comparing Cancer Surgery to Benign Surgery Patients: the Plot of Hazard Ratios and Corresponding 95% Confidence Intervals for Pulmonary Embolism and Deep Vein Thrombosis After the Discharge From the Hospital, by Cancer Type

Notes: Y-axis presented in logarithmic scale and truncated at 64 if hazard ratios or upper confidence intervals exceeded 64. All models adjusted for the matching variables, other major surgery, comorbidities (ischemic heart disease, peripheral vascular disease, cardiac arrhythmia, cerebrovascular disease, congestive heart failure, paralysis, hypertension, diabetes mellitus, chronic pulmonary disease, renal disease, and anemia) and length of index hospitalization.

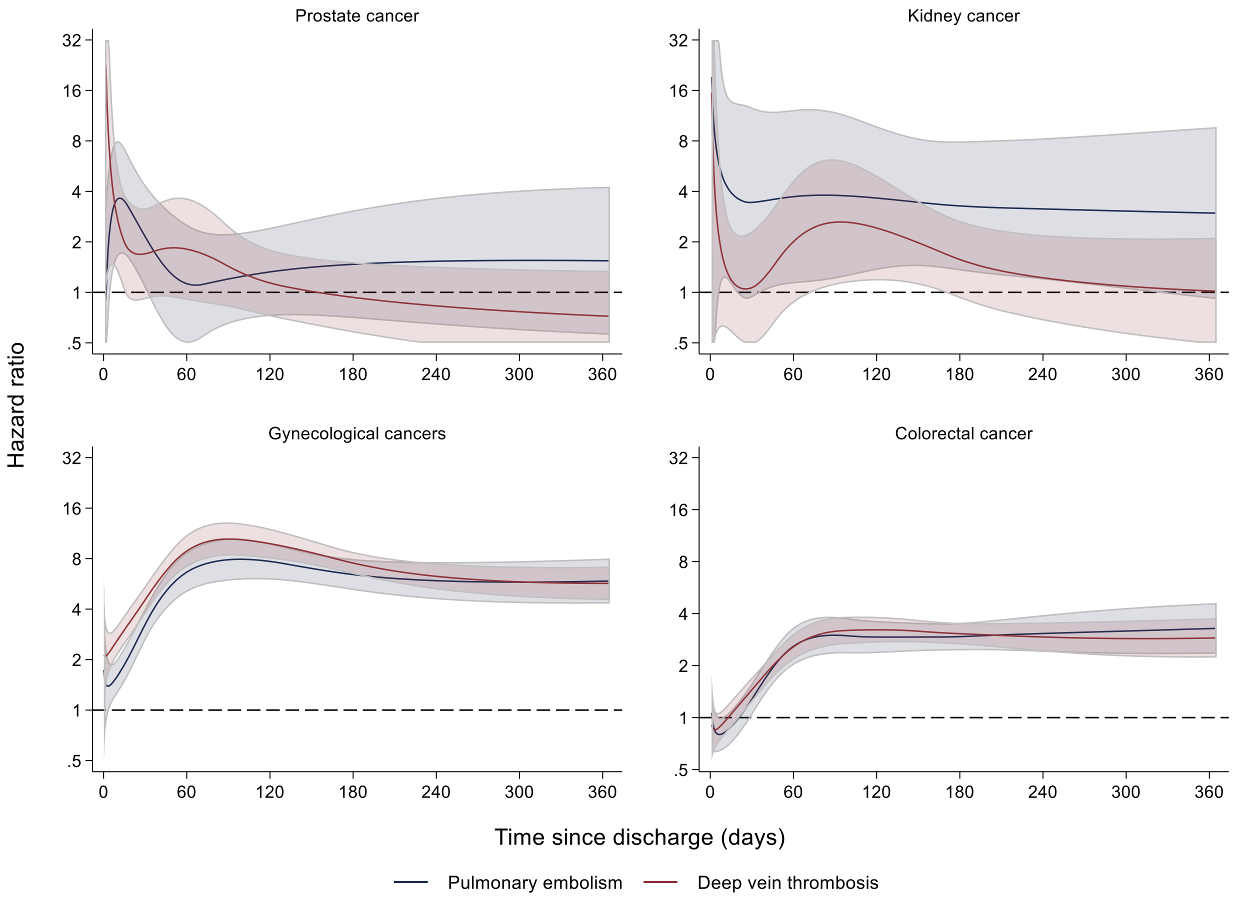

© 2024 Björklund J et al. *JAMA Network Open.*
