# Supplementary material for: Risk of Venous Thromboembolic Events After Surgery for Cancer
Source: JAMA Netw Open. 2024 Feb 2;7(2):e2354352. doi: 10.1001/jamanetworkopen.2023.54352 (PMC10837742; doi:10.1001/jamanetworkopen.2023.54352)
Supplement: Supplement 2. — Data Sharing Statement [file jamanetwopen-e2354352-s002.pdf]

Data Sharing Statement

Björklund. Risk of Venous Thromboembolic Events After Surgery for Cancer. *JAMA Netw Open*. Published February 02, 2024. doi:10.1001/jamanetworkopen.2023.54352

Data
Data available: No

Additional Information
Explanation for why data not available: Data sharing is not possible as according with the agreement with the provider of the data, the Swedish National Board of Health and Welfare. However, anyone can apply for extraction of the data from the Swedish National Board of Health and Welfare.
